# A range-wide synthesis and timeline for phylogeographic events in the red fox (*Vulpes vulpes*) 

**DOI:** 10.1186/1471-2148-13-114

**Published:** 2013-06-05

**Authors:** Verena E Kutschera, Nicolas Lecomte, Axel Janke, Nuria Selva, Alexander A Sokolov, Timm Haun, Katharina Steyer, Carsten Nowak, Frank Hailer

**Affiliations:** 1 Biodiversity and Climate Research Centre (BiK-F), Senckenberg Gesellschaft für Naturforschung, Senckenberganlage 25, 60325 Frankfurt am Main, Germany; 2 Department of Arctic and Marine Biology, University of Tromsø, N-9037 Tromsø, Norway; 3 Department of Environment, Government of Nunavut, X0A0L0 Igloolik, Canada; 4 Institute for Ecology, Evolution and Diversity, Goethe University Frankfurt, Max-von-Laue-Straße 13, 60438 Frankfurt am Main, Germany; 5 Institute of Nature Conservation, Polish Academy of Sciences, Mickiewicza 33, 31-120 Krakow, Poland; 6 Ecological Research Station of the Institute of Plant and Animal Ecology, Russian Academy of Sciences, Labytnangi, 629400, Russia; 7 Conservation Genetics Group, Senckenberg Research Institute and Natural History Museum Frankfurt, Clamecystraße 12, 63571 Gelnhausen, Germany

**Keywords:** Carnivores, Divergence time estimate, Generalist, mtDNA control region, Phylogeography, *Vulpes*

## Abstract

**Background:**

Many boreo-temperate mammals have a Pleistocene fossil record throughout Eurasia and North America, but only few have a contemporary distribution that spans this large area. Examples of Holarctic-distributed carnivores are the brown bear, grey wolf, and red fox, all three ecological generalists with large dispersal capacity and a high adaptive flexibility. While the two former have been examined extensively across their ranges, no phylogeographic study of the red fox has been conducted across its entire Holarctic range. Moreover, no study included samples from central Asia, leaving a large sampling gap in the middle of the Eurasian landmass.

**Results:**

Here we provide the first mitochondrial DNA sequence data of red foxes from central Asia (Siberia), and new sequences from several European populations. In a range-wide synthesis of 729 red fox mitochondrial control region sequences, including 677 previously published and 52 newly obtained sequences, this manuscript describes the pattern and timing of major phylogeographic events in red foxes, using a Bayesian coalescence approach with multiple fossil tip and root calibration points. In a 335 bp alignment we found in total 175 unique haplotypes. All newly sequenced individuals belonged to the previously described Holarctic lineage. Our analyses confirmed the presence of three Nearctic- and two Japan-restricted lineages that were formed since the Mid/Late Pleistocene.

**Conclusions:**

The phylogeographic history of red foxes is highly similar to that previously described for grey wolves and brown bears, indicating that climatic fluctuations and habitat changes since the Pleistocene had similar effects on these highly mobile generalist species. All three species originally diversified in Eurasia and later colonized North America and Japan. North American lineages persisted through the last glacial maximum south of the ice sheets, meeting more recent colonizers from Beringia during postglacial expansion into the northern Nearctic. Both brown bears and red foxes colonized Japan’s northern island Hokkaido at least three times, all lineages being most closely related to different mainland lineages. Red foxes, grey wolves, and brown bears thus represent an interesting case where species that occupy similar ecological niches also exhibit similar phylogeographic histories.

## Background

While current population genetic structuring tends to be weak in arctic mammalian specialists [1,2], species in temperate regions usually show more pronounced structuring [3-5], due to their survival in different refugia during the last glacial maximum (LGM) (reviewed in [6,7]). An exception to this trend is observed in some temperate-zone species with generalist habitat requirements and high mobility, like large carnivores that occur across a wide habitat and climatic gradient. Indeed, grey wolves (*Canis lupus*) and brown bears (*Ursus arctos*) show an overall weak phylogeographic structure with several widely distributed lineages [8-15].

Similar to the brown bear and the grey wolf, the red fox (*Vulpes vulpes*) is distributed across all northern continents (Europe, Asia, and North America), being the most widely distributed carnivore in the world [16]. The high mobility and adaptability of the red fox to different habitats and climates is reflected by its earliest appearance in the fossil record outside the southern refugia shortly after the last glacial maximum (LGM). Some 13,500 years before present, for instance, the red fox re-appeared in Northern Germany close to the ice sheets [17]. Daily distances of more than 10 km are common [18,19], and the longest recorded distance covered by a red fox was 302 km within less than a year’s time [20].

**Figure 1:**
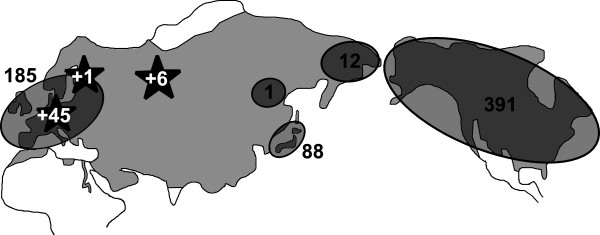
**Map of sample locations for all sequences used in this study (published data and newly generated data).** Current distribution range of the red fox [84] is shown in light grey. Black stars with white numbers indicate sampling locations for new data generated in this study. Circles indicate sampling regions from previous studies, with black numbers denoting numbers of published sequences for these regions. Details on all sequences used in this study are given in Additional file 1.

Although red fox phylogeography using DNA sequence data from a ca. 268 – 342 bp fragment of the mitochondrial (mt) control region has been investigated before [21-27], these studies had a regional focus or sampling gaps in Siberia and Asia (Figure 1). In addition, so far the published data has not been collated to investigate range-wide processes, and no timeline has yet been established for phylogeographic events. Previous studies described two major red fox lineages – one with a Holarctic distribution and a Nearctic lineage consisting of three sublineages (widespread lineage, eastern lineage, mountain lineage) [23,24,27]. A study on Japanese red foxes found two main lineages in Japan: one lineage that was exclusively found on Japan’s northern island Hokkaido (Hokkaido II), and another lineage that comprised three Japanese sublineages occurring on Hokkaido and on Japan’s main southern islands Honshu and Kyushu (Hokkaido Ia, Hokkaido Ib, Honshu/Kyushu) along with mainland Asian red foxes [21].

We here present novel data from red foxes of various Eurasian populations, notably including individuals from central Siberia, a biogeographically important region with the potential to link European with East Asian and/or North American populations. In a range-wide synthesis of published and publicly available control region sequences combined with newly generated data (Figure 1), the identity and geographic distribution of previously described lineages is validated. Based on this novel assessment of mtDNA structure in red foxes, we establish a timeline of major phylogeographic events using a Bayesian coalescence approach with multiple fossil tip and root calibration points. We compare these results for red foxes to previously published findings from other carnivores with a Holarctic distribution, allowing us to identify common phylogeographic patterns and processes.

## Results

In our analysis of 729 red foxes (a map with the geographic locations is given in Figure 1 and a complete compilation is found in Additional file 1), we found 95 variable sites that defined 175 haplotypes in a 335 bp-alignment of the mitochondrial control region (Table 1). Haplotype diversity for the whole dataset was 0.948 +/− 0.005, and nucleotide diversity was 0.057 +/− 0.028 (Table 1). The 52 newly sequenced individuals from Siberia, Germany, Poland, and Finland formed 25 haplotypes. Most of these haplotypes (n = 22) had not been encountered in previous studies (Additional file 2). All newly obtained sequences have been submitted to the EMBL database [EMBL:HF677203-HF677255].

**Table 1:** Summary statistics of genetic variability of major red fox mtDNA control region lineages

**Geographic region, mtDNA lineage**	**n**	**S**	**N**_**H**_	**Hd**	**π**	**Fu’s F**_**S**_
All samples	729	95	175	0.948 +/− 0.005	0.057 +/− 0.028	−23.363***
Holarctic lineage	405	67	131	0.964 +/− 0.004	0.047 +/− 0.024	−23.701***
Japan, Hokkaido II	6	3	3	0.600 +/− 0.215	0.003 +/− 0.003	−0.189*
Japan, Honshu/Kyushu	29	5	6	0.517 +/− 0.106	0.003 +/− 0.003	−1.295**
North America, Nearctic lineage	289	34	35	0.742 +/− 0.026	0.019 +/− 0.010	−4.347***
North America, eastern lineage	72	9	8	0.678 +/− 0.050	0.003 +/− 0.002	−2.423**
North America, mountain lineage	186	20	18	0.429 +/− 0.046	0.006 +/− 0.004	−6.295***
North America, widespread lineage	31	13	9	0.847 +/− 0.036	0.022 +/− 0.012	2.886

*n* sample size (number of individuals), *S* number of segregating sites, *N*_*H*_ number of distinct haplotypes, *Hd* haplotype diversity, *π* nucleotide diversity, and *Fu’s* F_S_, an indicator of population expansion (when negative and significant). Asterisks indicate significance level (*p ≤ 0.02; **p ≤ 0.01; ***p ≤ 0.001).

### Major mitochondrial clades and population structuring

**Figure 2:**
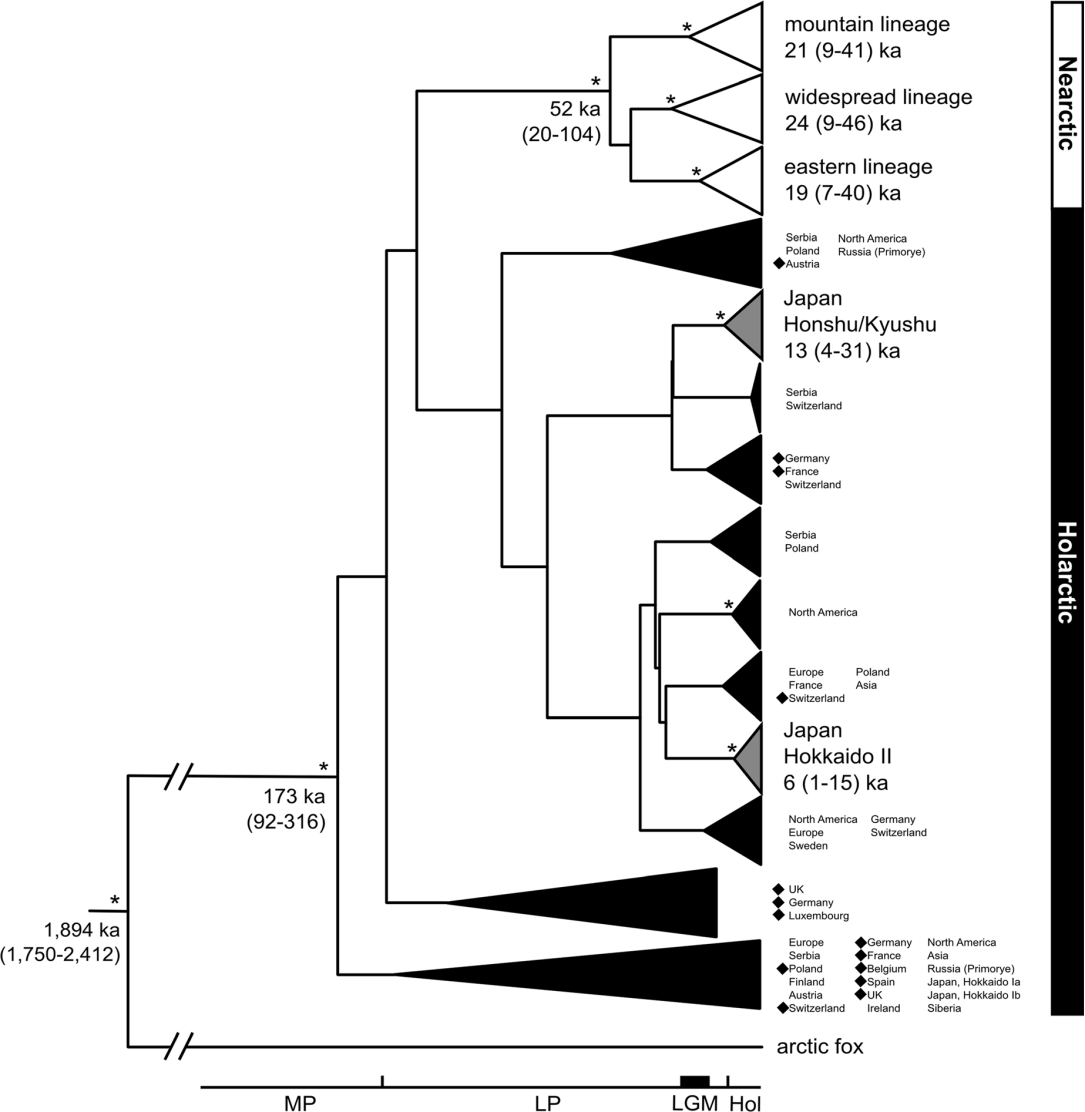
**Maximum clade credibility tree with time estimates for colonization events and basal diversifications within red fox mtDNA control region lineages.** For the dating of phylogeographic events we used a combined approach, utilizing tip dates based on published ancient DNA red fox sequences [25], plus the arctic fox as exterior calibration point. This tree shows the results for a root height prior of 1.75-4 million years (My), according to the 95% credibility interval in Perini et al. [80] for the divergence time between red and arctic fox (Table 2). White: Nearctic lineage haplotypes; grey: Japanese haplotypes (lineages Honshu/Kyushu and Hokkaido II); black: Holarctic lineage haplotypes, including Japanese lineages Hokkaido Ia and Ib; ka: thousand years. Nodes marked with an asterisk were supported by posterior probability values >0.95. Samples used for tip calibration are marked with a ♦ symbol. Median ages and 95% highest posterior density ranges in brackets show the estimated ages of major lineages, and of the most basal nodes within these lineages. Our discussion focuses on lineages/nodes with ≥0.95 statistical support, recognizing that longer mtDNA sequences will be required to resolve larger proportions of the red fox mitochondrial phylogeny (see [58-62]).

**Figure 3:**
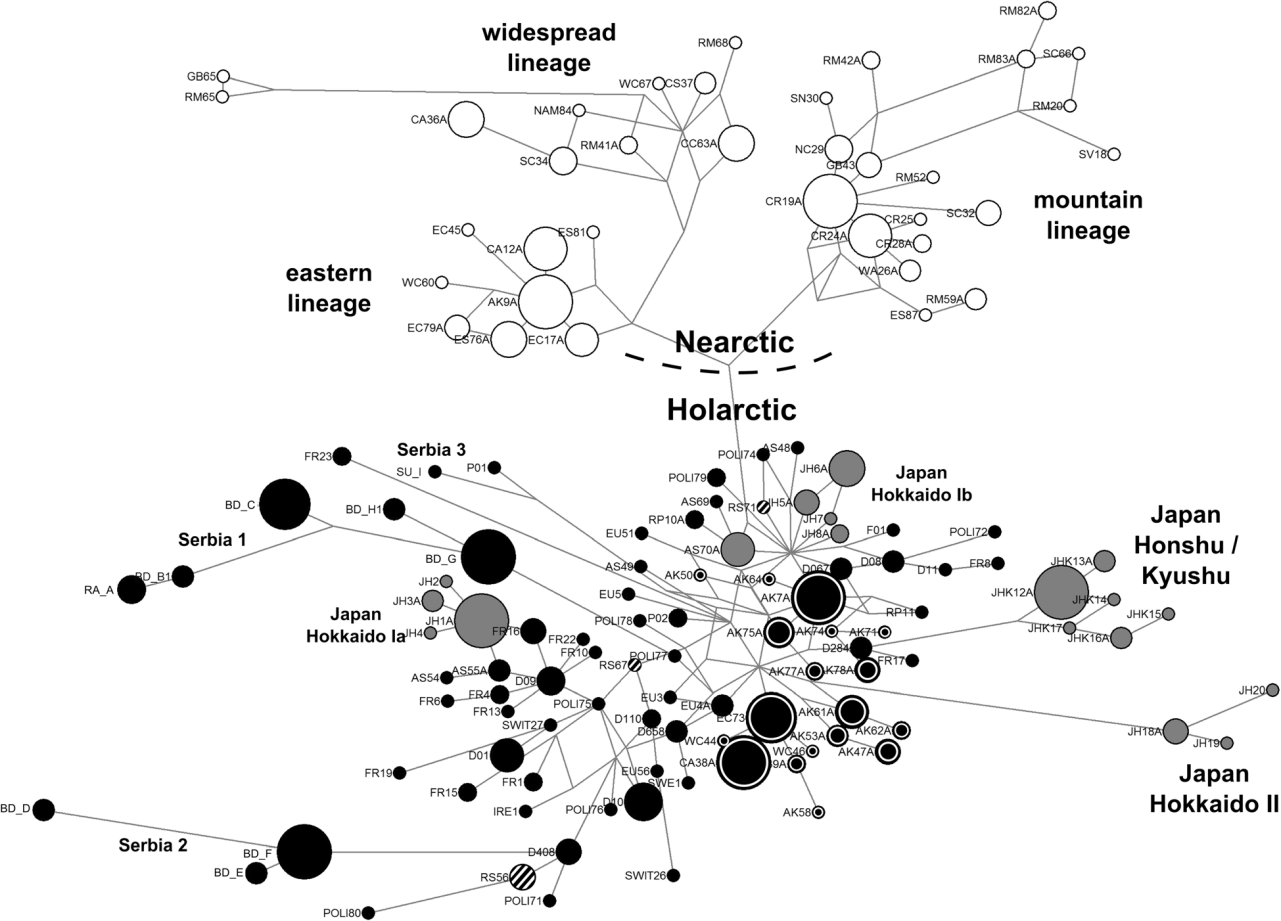
**Median joining network of genetic variation at a 335 bp fragment of the mitochondrial control region in 693 red foxes.** 35 ancient samples from Teacher et al. [25] and one modern sample from Valière et al. [28] were excluded due to their shorter sequence length. White: Nearctic lineage haplotypes; grey: Japanese haplotypes (all Japanese lineages); black: Holarctic lineage haplotypes, except for North American, Japanese and central Siberian haplotypes; black with white circle: North American haplotypes (Holarctic lineage); black with white stripes: central Siberian haplotypes (Holarctic lineage).

We confirmed all previously described lineages (Figures 2 and 3, Additional file 3). All Nearctic lineages and the Japanese Hokkaido II and Honshu/Kyushu lineages were distinct in a median-joining network (Figure 3), although some received less than 95% posterior support in the BEAST analysis (Figure 2). However, the most basal split within each of these lineages received high posterior support in the BEAST analysis (Figure 2). A phylogenetic analysis of haplotype data conducted in MrBayes recovered a tree with a topology congruent with the tree obtained from BEAST (Additional file 3), in accordance with previous lineage definitions. Therefore, despite some uncertainty with regard to their placement in the red fox mtDNA phylogeny, those regional lineages represent distinct clades that capture aspects of the evolutionary history of red foxes. Our analysis and discussion of regional lineages therefore focuses on groupings with high statistical support and/or lineages that were specifically defined in previous studies.

Nucleotide and haplotype diversity were highest for geographically widely distributed lineages (Holarctic, Nearctic, and Nearctic widespread lineages), and lowest for regionally restricted lineages (Japanese Hokkaido II and Honshu/Kyushu lineages, Nearctic mountain and eastern lineages) (Table 1).

North American red foxes fell into four mitochondrial groups: a Nearctic lineage that comprised three sublineages (mountain, eastern, and widespread lineages), and a Holarctic lineage that was composed of North American and Eurasian red foxes (Figures 2 and 3). These lineages were previously described and discussed in detail by Aubry et al. [23], Sacks et al. [24], and Statham et al. [27].

All Eurasian red foxes were placed in the Holarctic lineage (Figures 2 and 3). In contrast to the Nearctic lineage, most support values for phylogenetic groupings within the Holarctic lineage were low: no geographically restricted sublineages in the Holarctic lineage received high statistical support, except for two sublineages from Japan (Figures 2 and 3; and see below). Haplotypes from other geographic regions did not form monophyletic groups, but were scattered across the Holarctic lineage (Figures 2 and 3). However, the few instances of haplotype sharing occurred only between geographically neighboring populations (e.g., Germany, Switzerland, France; see Additional file 1). This weak phylogeographic structuring within the Holarctic lineage was consistent with previous findings by Teacher et al. [25] and Edwards et al. [26], who discussed red fox phylogeographic patterns in Europe more in detail.

All 52 newly sequenced red foxes from Siberia, Germany, Finland, and Poland belonged to the Holarctic lineage (Figures 2 and 3). The six central Siberian individuals formed three not previously described haplotypes that were not especially closely related to each other (Figure 3, Additional files 1 and 2). Also the Finnish red fox carried a novel haplotype (F01). The thirty newly sequenced German individuals formed ten haplotypes. Seven of these were new; three haplotypes were identical to previously published sequences from French, Swiss or other European populations [23,25,28] (Additional files 1 and 2). One German haplotype (individuals D408, D655, and D660) was shared with a Polish red fox (POLI68) from the present study. The newly sequenced Polish red foxes formed thirteen haplotypes (Figure 3, Additional files 1 and 2), twelve of which had not been described previously. Besides the haplotype described above (POLI68), another was identical to a German haplotype from the present study (D08) and to a Swiss haplotype from a previous study (Swit12) [25,28] (Additional file 1). One Polish haplotype was closely related to a Serbian haplotype (I) that was previously described as being distinct from all other Serbian red foxes [22] (Serbia 3; see Figure 3).

The other Serbian red foxes clustered separately into two groups that were distinct from each other (Serbia 1 and Serbia 2) within the Holarctic lineage, confirming Kirschning et al. [22]. In contrast to Kirschning et al. [22] findings, however, the two groups were separated by several Eurasian haplotypes and distinct from the rest of the Holarctic diversity in the median-joining network (Figure 3). In the Bayesian inference tree (Additional file 3), all three Serbian lineages received less than 95% posterior support.

The Japanese samples formed four distinct groups that fell within the Holarctic diversity (Figures 2 and 3, Additional file 3). These Japanese lineages were separated by intermediate haplotypes found on the Eurasian mainland (Figure 3). Unlike in previous analyses [21,29], our extended sampling and range-wide synthesis revealed that the Honshu/Kyushu lineage was not closely related to Hokkaido Ia, Hokkaido Ib, and Asian mainland red foxes, but formed a distinct lineage (Figures 2 and 3, Additional file 3). Red foxes from the northern island Hokkaido formed three separate groups. The Hokkaido II lineage remained clearly distinct from the other red foxes, as originally described by Inoue et al. [21] and confirmed using mitochondrial cytochrome b data by Yu et al. [29] (Figures 2 and 3). Hokkaido I red foxes clustered into two subgroups in the Holarctic lineage (Figures 2 and 3) consistent with Inoue et al. [21] findings of sublineages Hokkaido Ia and Hokkaido Ib. In contrast, however, our range-wide synthesis recovered Eurasian mainland haplotypes that separated the Hokkaido Ia and Ib groups from each other (Figure 3). Further, one haplotype from Hokkaido Ib (JH9) was identical to a haplotype found by Aubry et al. [23] on the Asian mainland (AS70, see Figure 3 and Additional file 1), supporting the close relatedness between these populations.

### Timing of phylogeographic events in the red fox

The influence of different root heights on the divergence time estimate for the red fox from the arctic fox (*Vulpes lagopus*) was evaluated by performing different simulations in BEAST. However, each BEAST run converged on the youngest time frame allowed by the prior for the speciation event (i.e., close to the different minimum root heights of 1.75 million years (My) and 5.1 My, respectively; see Table 2). Similarly, a BEAST run that only applied tip dating (i.e., with no constraint on minimum root height) yielded a divergence time estimate for red/arctic foxes of 301 (101–611) thousand years (ka). This time estimate is less than the first appearance of the red fox in the fossil record, ca. 0.5 – 1 million years ago (Mya) [30,31], reinforcing the value of mixed tip/root calibration approaches in BEAST [13,32]. Hence, our data do not allow an accurate assessment of the divergence time between red and arctic foxes - the focus of this paper is on much more recent phylogeographic events within red foxes.

**Table 2:** Comparison of BEAST dating results employing a relaxed-clock approach with combined tip (interior) and root (exterior) calibration

**Scenario**	**Root height prior (min.) [ka]**	**Median posterior substitution rate ** **[per site and 10**^**6**^ **years]**	**Divergence time estimates [ka]**							
			**Red / arctic fox**	**Red fox diversification**	**Nearctic lineage**	**North America, eastern lineage**	**North America, widespread lineage**	**North America, mountain lineage**	**Japan, Honshu / Kyushu**	**Japan, Hokkaido II**
*Scenario 1*	1750	33.2%	1,894 (1,750–2,412)	173 (92–316)	68 (39–93)^a^	42 (18–82)^a^	42 (18–82)^a^	52 (20–104)	24 (9–52)^a^	27 (11–60)^a^
*Scenario 2*	5100	25.6%	5,325 (5,100–5,847)	235 (118–423)	128 (73–206)^a^	57 (23–121)^a^	73 (28–164)	57 (23–121)^a^	64 (46–94)^a^	45 (22–69)^a^
*Scenario 3*	500	41.9%	586 (500–874)	129 (78–208)	61 (30–92)^a^	32 (13–61)^a^	32 (13–61)^a^	39 (14–74)	21 (8–51)^a^	32 (13–64)^a^

All three scenarios were using the same tip dates, but varying root heights as exterior calibration points (red/arctic fox divergence time estimate). *Scenario 1:* uniform prior for root height of 1.75-4 million years (My) according to the 95% credibility interval in Perini et al. [80] (Figure 2). *Scenario 2:* uniform prior for the root height of 5.1-6 My according to the 95% credibility interval in Nyakatura and Bininda-Emonds [81]. *Scenario 3:* lognormal root height prior based on the first appearance of the red fox in the fossil record (0.5-1 My ago) [30,31], setting the minimum age of the root height to 0.5 My. The 95% interval of the lognormal prior included a period of up to 5.9 My.

^a^Less than 95% posterior support for the divergence from the next most closely related sequence, but at least 95% support for the most basal divergence within the lineage. Note also that, despite uncertainty regarding the phylogenetic placement of these groups, their inferred age was relatively similar across calibration scenarios.

*ka* thousand years.

Importantly, our analyses in BEAST indicated that this uncertainty about the root height of the tree (i.e., the divergence from arctic foxes) did not have a major effect on the dating of evolutionary events within red foxes. Comparing the BEAST simulations with a root height of minimum 0.5 My to the simulation setting it to at least 5.1 My – the two most extreme scenarios including a root height and tip dating – the estimated divergence time for the red/arctic fox speciation event varied by a factor of 10, whereas the median time estimates for phylogeographic events within red foxes varied only by a factor of 1.4-3, with overlapping confidence intervals (Table 2). It thus appeared that the inconsistent root height (red/arctic fox divergence time) only slightly impacted our time estimates for phylogeographic events *within* red foxes, which is likely in part due to our additional use of interior tip calibrations based on ancient DNA sequences from known-age fossil remains.

BEAST runs yielded posterior substitution rate estimates of 33.2% for *Scenario 1* (with a root height of 1.75 My), and 25.6% or 41.9% for *Scenarios 2* and *3*, respectively (Table 2). A recent study of red fox mtDNA obtained a similar mutation rate estimate (ca. 26.29-33.81%, depending on whether the substitution model included a gamma correction; Edwards et al. [26]). It is interesting to note that these results [26] were based on a partial control region fragment combined with a partial cytochrome b fragment, precluding direct comparisons of the estimates. Similar reasoning applies to the mutation rate estimate (28.8%) obtained for mtDNA in brown and cave bears [33], that is based on a fragment that only overlaps partially with our alignment.

Due to low posterior support values for many internal nodes (Figure 2 and Additional file 3), our analyses do not allow to identify the next most closely related sequence of some of the described lineages. However, regardless of their exact phylogenetic placement, estimated divergence times of regional lineages from their most closely related sequence remained largely constant (Table 2).

**Figure 4:**
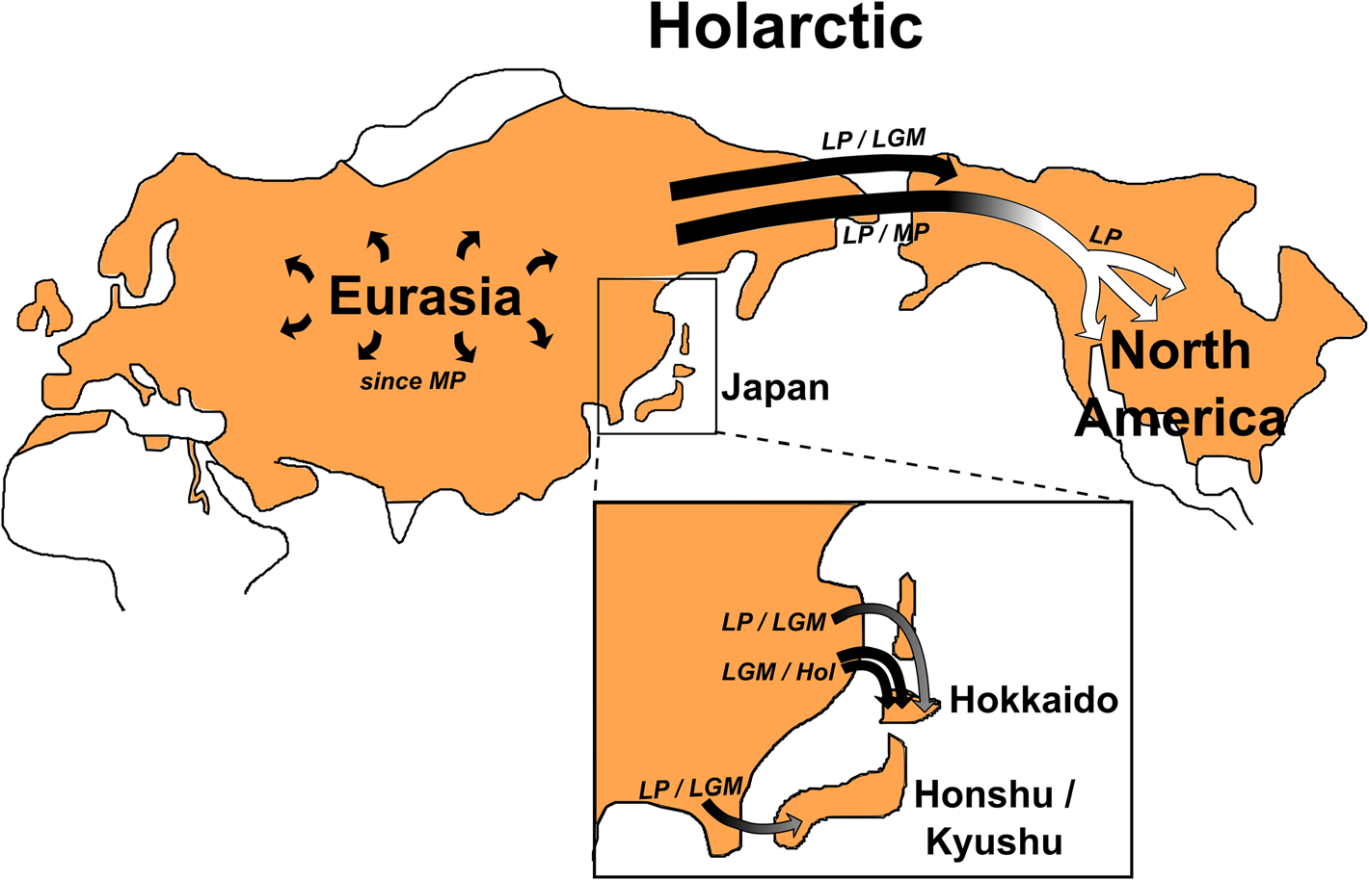
**Map outlining major phylogeographic events in red foxes as reconstructed using a Bayesian coalescence approach with multiple fossil tip and root calibration points.** Current distribution range [84] is shown in foxy red. White: Nearctic lineage; grey: Japanese lineages; black: Holarctic lineage (excluding Japan-restricted lineages). MP: Mid Pleistocene, LP: Late Pleistocene, LGM: Last Glacial Maximum, Hol: Holocene. Note that not all sublineages within the Holarctic lineage are currently distributed across Eurasia and North America; only some lineages show extensive range expansions (Figures 2 and 3, and Additional file 3).

Based on the most basal divergences among extant lineages (Figure 2, Table 2), red foxes started to diversify in Eurasia during the end of the Mid Pleistocene (Figure 4). North America was colonized independently by several lineages from this Holarctic diversity (Figures 2 and 4, Table 2), as indicated by simulations in BEAST. The colonization event forming the Nearctic lineage happened around the Mid or Late Pleistocene (Figures 2 and 4, Table 2). All three Nearctic sublineages (eastern, mountain, and widespread) were formed before the LGM (Figures 2 and 4, Table 2). The close relationship between North American and Eurasian Holarctic lineage red foxes (Figures 2 and 3) indicated that the North American Holarctic lineage colonized North America much more recently than the Nearctic lineage, probably around the LGM (Figure 4).

Also Japan was colonized several times independently by individuals from the Holarctic lineage. The southern islands Honshu and Kyushu were colonized during the Late Pleistocene, or around the LGM (Figures 2 and 4, Table 2). The northern island Hokkaido was colonized several times. The lineage Hokkaido II arrived most likely during the end of the Late Pleistocene (Figures 2 and 4, Table 2). Due to the close relationship of Hokkaido Ia and Hokkaido Ib red foxes to Eurasian mainland red foxes (Figures 2 and 3), the two groups most likely colonized Hokkaido more recently than the Hokkaido II lineage did, probably around the LGM (Figure 4).

### Demography

When we tested the exponential population growth model in BEAST, the exponential growth rate fluctuated around zero, so we could not reject the constant population size model for the entire dataset. Fu’s F_S_ showed a signal of population growth across all red fox sequences, with a highly significant (p ≤ 0.001) value of −23.363 (Table 1). Except for the Nearctic widespread lineage, all major lineages had a significant (p ≤ 0.02) F_S_, with negative values ranging from −0.189 for the Japanese Hokkaido II lineage to −23.701 for the Holarctic lineage (Table 1). Star-like structures in a median joining network (Figure 3) also indicated evolutionarily recent population growth for these lineages.

We calculated mismatch distributions for all lineages in Arlequin. For the Nearctic lineage, the Nearctic eastern and widespread lineages, and the Japanese Hokkaido II lineage, population growth was confirmed by mismatch distribution analyses, where simulations in Arlequin did not differ significantly (p > 0.05) from expectations under the sudden expansion model (Table 3). The Nearctic lineage started to diversify around the Late Pleistocene/LGM, as further supported by our dating in BEAST of the most basal bifurcation within each lineage (Figure 2, Table 2 and 3). The estimated sudden expansion for the Nearctic eastern lineage of 23 (11–35) ka (assuming 7.1% per-lineage substitution rate per My), and 5 (2–7) ka (assuming 33.2% per-lineage substitution rate per My) (Table 3) overlapped with the period indicated by the BEAST analyses (Figure 2, Table 2). Population expansion of the Japanese Hokkaido II lineage started about 36 (0–85) ka with 7.1% per-lineage substitution rate per My, and about 8 (0–18) ka with 33.2% per-lineage substitution rate per My (Table 3), similar to the date estimate from the BEAST analysis of 6 (1–15) ka (Figure 2). The very recent date estimates for the most basal bifurcation in Hokkaido II during the Holocene (Figure 2, Table 3) were possibly a result of genetic drift removing more ancient lineages in this clade (e.g., [34]). Because deviation from the sudden expansion model in Arlequin was significant for the remaining major clades, we could not determine the timing for any onset of population expansion for them. However, based on the BEAST results, the Honshu/Kyushu population diversity was slightly older than the Hokkaido II population diversity, with 95% credibility intervals spanning the LGM (Figure 2, Table 3).

**Table 3:** Mismatch distribution analysis under a sudden expansion model and time since expansion calculated for different mitochondrial lineages

**Geographic region, mtDNA lineage**^**a**^	**τ (confidence interval)**	**Deviation from sudden expansion model (p-value)**	**Time since expansion [ka] (7.1% substitutions/lineage/My)**^**b**^	**Time since expansion [ka] (33.2% substitutions/lineage/My)**^**b**^
North America, Nearctic lineage	10.580 (0.541–19.053)	0.115	222.4 (11.4–400.5)	47.6 (2.4–85.7)
North America, eastern lineage	1.086 (0.516–1.641)	0.371	22.8 (10.8–34.5)	4.9 (2.3–7.4)
North America, widespread lineage	13.295 (0.697–24.838)	0.071	279.5 (14.7–522.1)	59.8 (3.1–111.7)
Japan, Hokkaido II	1.725 (0–4.025)	0.743	36.2 (0–84.6)	7.8 (0–18.1)

^a^Mismatch analyses were performed for all lineages (see Table 1). Time since expansion was only calculated for lineages where τ did not differ significantly from the sudden expansion model (p > 0.05).

^b^Time estimates calculated based on a per-lineage substitution rate of 7.1% (u = 2.379*10^-5^; see [2]) or 33.2% per million years (u = 1.112*10^-4^; see Table 2), respectively.

*ka* thousand years, *My* million years.

The two methods we used to detect population growth are characterized by different levels of sensitivity: tests based on mismatch distribution analyses are conservative and less powerful than Fu’s F_S_ to detect population growth [35]. This might explain inconsistencies in our demographic inferences: Fu’s F_S_ was negative and significant for the Nearctic mountain lineage and the Japanese Honshu/Kyushu lineage (Table 1), whereas mismatch distribution analyses failed to detect population growth (thus not enabling us to obtain an estimate of the timing from the mismatch distribution analysis for these lineages). The Nearctic widespread lineage showed the opposite result: while mismatch distribution analyses indicated population growth (Table 3), Fu’s F_S_ was positive (Table 1). Our inferred timing of the onset of sudden expansion of this lineage from mismatch distribution analysis (about 280 (15–522) ka with 7.1% per-lineage substitution rate per My, and about 60 (3–112) ka with 33.2% per-lineage substitution rate per My; Table 3) should therefore be taken with caution, especially since the time estimate inferred from BEAST (24 (9–46) ka; Figure 2; see also Table 2) was younger.

## Discussion

Our synthesis of 677 published and publicly available sequences together with 52 newly obtained sequences includes previously unsampled geographic regions (e.g., central Siberia; Figure 1), and confirms previous classifications of mitochondrial lineages in red foxes [21,23,24,27]. In this study, we delineate the range-wide timing and pattern of phylogeographic events for this widespread carnivore. During the Mid Pleistocene, a period characterized by repeated climatic oscillations, red foxes started to diversify. One lineage (the Holarctic lineage) today occurs across most of the entire distribution range, including North America, Europe, and Asia. In contrast, other red fox lineages are regionally restricted. During the Late Pleistocene and Holocene, North America and Japan were colonized several times independently by red foxes (Figures 2 and 4), likely at times when landbridges connected these islands to adjacent landmasses (except for Honshu/Kyushu, see below). Our dating of subsequent diversification events suggests that demographic expansions in many red fox populations occurred since the Late Pleistocene. Our suggested timeline and pattern of phylogeographic events in red foxes closely resembles the scenarios described for the ecologically similar and co-distributed grey wolves and brown bears [8,10,13,36], reinforcing that ecological and climatic factors had similar effects on temperate zone species.

### Phylogeographic history of red foxes

#### Mid Pleistocene

According to the fossil record [37], red foxes were already present in Eurasia during the Mid Pleistocene, around 300 ka, a timing consistent with our finding of the most basal diversification within red foxes during that time (Figure 2). For extended time periods since the Mid Pleistocene, North America was connected to Eurasia via the Bering landbridge [38], at glacial times of lower sea level, allowing species like the red fox to colonize North America several times independently. Red fox fossil remains from Alaska indicate that North America was first colonized during the Illinoian glaciation (ca. 300–130 ka) [39]. This period overlaps with our estimate for the emergence of the Nearctic lineage (*Scenario 2*, Table 2), likely corresponding to the colonization of North America from Eurasia around the end of the Mid Pleistocene (Figures 2 and 4).

#### Late Pleistocene

The fossil record indicates that some North American red foxes persisted through the Late Pleistocene glaciations (Wisconsin; ca. 100–10 ka) south of the ice sheets [40-42]. It would be interesting to use ancient DNA techniques to verify whether such remains indeed belong to the Nearctic red fox mitochondrial lineage. Consistent across a broad range of different rate calibrations (Table 2), we found a Late Pleistocene diversification of the Nearctic lineage into three sublineages (Figures 2 and 4), the eastern, mountain, and the widespread lineages.

During the same time frame, the Holarctic lineage further diversified in Eurasia (Figures 2 and 4). From this diversity, Japan was colonized several times independently. Japan’s main southern islands Honshu and Kyushu have been isolated from the Eurasian mainland and Hokkaido in the north since the Mid Pleistocene (reviewed in [43]). The Tsugaru Strait separating Hokkaido from Honshu/Kyushu represents a biogeographic barrier (Blakiston’s line [44]) for many species since the Mid Pleistocene (reviewed in [43]), including the red fox. However, red foxes appear to have colonized Honshu and Kyushu during the Late Pleistocene (Figures 2 and 4). The European fossil record indicates potential human influence on red fox dispersal (based on the common finding of red fox remains in European archaeological assemblages; [17]). Humans first reached Japan ca. 50 ka (reviewed in [45]), consistent with our red fox colonization time estimate for Honshu and Kyushu. Red foxes might thus have reached these islands through human introduction, although non-human facilitated rafting or temporary ice bridges connecting Honshu and Hokkaido [46] are also plausible alternatives.

During most of the Late Pleistocene glacial phases, Hokkaido was repeatedly connected to the Eurasian mainland via the Russian island Sakhalin [43]. Indeed, red foxes from the Hokkaido II lineage colonized Hokkaido during the Late Pleistocene (Figures 2 and 4), most likely from the mainland via these northern landbridges.

#### Around the last glacial maximum (LGM)

The phylogeographic structure found in North American red foxes has been interpreted to result from range fragmentation by ice sheets during glacial maxima [23]. In contrast, we did not find a strong signal of red fox survival during the LGM in distinct and isolated southern refugia in Eurasia. In fact, the Holarctic lineage appears to be geographically largely unstructured and shows only weak signs of past population fragmentation (Figures 2 and 3; see also [25,26]). This could support the notion of red foxes as habitat generalists that were able to survive the LGM period in the vast arctic steppes, as shown for a now-extinct wolf ecotype [34]. However, red fox remains have not been found in the arctic steppe regions of northern and central Europe during the LGM [17]. Indeed, contemporary red fox distribution and population ecological studies suggest that the species has a northern (and upper latitudinal) limit in its distribution, where more arctic-adapted species like the arctic fox have a competitive advantage (e.g., [47]). Survival of red foxes during the LGM in southern refugia is supported by several lines of evidence: (i) our finding of (albeit weakly) differentiated mitochondrial sublineages in Serbia (Figure 3), (ii) genetic differentiation of Iberian red foxes from other European populations [26], (iii) signs of low connectivity among different Mediterranean populations [48], and (iv) the restriction of red foxes to southern European refugial regions during the LGM [17]. Similar to findings from brown bears [10], the high dispersal capability of red foxes and their rapid recolonization of suitable habitats after deglaciation may have led to gene flow among refugia, preventing the development of pronounced phylogeographic structuring. Our finding of signals of population expansions in many red fox lineages (Tables 1 and 3) likely reflects this postglacial demographic growth.

The following conditions may therefore have prevented the formation of deeply separated mitochondrial lineages in Eurasian red foxes: only short periods (ca. 10 ka [17]) of geographic restriction in refugia for genetic differentiation, and potential for some gene flow among such refugial regions in periods of temporary warming within longer climatic cold phases. Ongoing range expansion of red foxes north and into higher altitudes into traditional arctic fox habitats (e.g., [49-51]) may mirror this situation of rapid recolonization of northern habitats.

The extensive mitochondrial gene flow among Eurasian red fox populations also reached parts of North America, forming a vast Holarctic population (Figure 4). Ice-free regions of Alaska and the Yukon (Beringia) were connected to Eurasia via the Bering landbridge during glacial maxima, but separated from regions south of the Laurentide and Cordilleran ice sheets [7]. Due to postglacial sea level rise, the Holarctic North American population was eventually isolated from the rest of the Holarctic lineage when the Bering landbridge was closed. Today, North American and Eurasian haplotypes from the Holarctic lineage are still intermingled (Figures 2 and 3), confirming their recent evolutionary separation. However, we found no haplotype sharing between North American and Eurasian red foxes (but note the shared haplotype between mainland Asia and Hokkaido; see Additional file 1), likely reflecting a post-LGM interruption of trans-Beringian gene flow. Similarly, the British Isles belonged to the Eurasian landmass during much of the Late Pleistocene and early Holocene. Edwards et al. [26] found red foxes from the British Isles to be only weakly differentiated from European main land foxes, resulting from recent postglacial isolation. Japan’s northern island Hokkaido was also repeatedly part of the Eurasian landmass during the Late Pleistocene, which is reflected by the presence of at least three distinct red fox lineages (Hokkaido Ia, Hokkaido Ib, and Hokkaido II; Figures 2 and 3; [21]). Hokkaido I haplotypes (Ia and Ib) are closely related to Eurasian mainland haplotypes (Figures 2 and 3), suggesting that they were isolated only recently from the rest of the Holarctic lineage. This may have occurred after the LGM (Figures 2 and 4) when rising sea levels isolated Hokkaido from the Asian mainland [43].

Genetic and paleontological data indicate that population expansion after the LGM occurred rapidly in European red foxes ( [17,26]; see also the signals of demographic expansion in Table 3). The finding of sublineages within the Holarctic diversity in southern Europe [26,48] indicates that some southern red fox populations contributed less to the postglacial recolonization of Eurasia, while other lineages showed wide-ranging dispersal, even across Beringia (Figure 4).

Despite these signals of extensive wide-ranging gene flow in red foxes, mtDNA also shows some signals of currently restricted gene flow, even within continents. Studies reporting local adaptations by red foxes in some regions that are discussed below confirm this view. In our dataset, the only instances of haplotype sharing occur among neighboring populations in Europe, and a single case involving Hokkaido and the Asian mainland (Additional file 1). Similarly, studies on red foxes using nuclear microsatellites have shown dispersal restrictions on shorter time scales [24,52-54], consistent with lower levels of gene flow today.

### Common phylogeographic trends in Holarctic carnivores

Two other large carnivores that have a Holarctic distribution and generalist habitat requirements are the brown bear and the grey wolf. Both species are highly mobile and flexible regarding their habitat requirements. Besides their ecology, they share remarkably similar phylogeographic patterns with the red fox. All three species are characterized by very widespread Holarctic mitochondrial lineages that are distributed across Eurasia and North America, and only some locally restricted lineages - indicating dispersal limitations in some regions of the Holarctic [8,9,11-13,34,55].

Independent colonization events from Eurasia led to the establishment of several endemic North American lineages such as grey wolves [34] and brown bears [13]. Those lineages survived the Late Pleistocene glaciations south of the North American ice sheets. Since the LGM period, additional Eurasian lineages immigrated via the Bering landbridge into the Nearctic, leading to current denomination of those lineages as Holarctic (Figure 4). Brown bears also inhabit the Japanese island Hokkaido. Similar to the situation in red foxes, Hokkaido was colonized at least three times by brown bears. Hokkaido harbors three distinct brown bear lineages, each being most closely related to different mainland lineages [56].

The grey wolf is another extensively studied example of a Holarctic generalist that displays a worldwide weak phylogeographic pattern, but with some geographically restricted mitochondrial lineages. It appears that especially some southern wolf populations did not expand after the Pleistocene glaciations: two distinct lineages survived south of the Himalayas [9]. In brown bears, a similar situation has been described for Syria and Iran [11,13], and in red foxes analogous evidence exists for Iberia [26,48] and Serbia (Figure 3; data from Kirschning et al. [22]).

Likely reflecting even more recent processes, grey wolves have been shown to be locally adapted to specific habitat and foraging conditions in Pacific temperate coastal rainforests [12,15]. Further, eastern European grey wolf population structure appears to be correlated with ecological factors [36,57]. As reviewed by Sacks et al. [24], North American montane red fox populations show physiological and morphological adaptations to cold climate, and are genetically distinct from other red fox populations (Nearctic mountain lineage) [23]. A refined sampling in this region supported an indigenous origin of the Sacramento Valley population, which differs in body size from the montane red fox populations [24].

In summary, bears, wolves, and red foxes show similar phylogeographic structuring and evidence of large-scale gene flow, but also of recently reduced levels of connectivity and local adaptations in some regions.

## Outlook

Fruitful future research will be a refined sampling in several geographic regions, especially in northern Africa, Asia, and Eastern Europe. Because some southern populations appear not to have contributed to large-scale postglacial range expansions, those seem particularly likely to harbor previously undetected genetic variation. High sequence variability in some mitochondrial genomic regions can provide enough information to detect phylogeographic events, especially the hypervariable 5′ end of the mammalian mtDNA control region. However, analysis of a larger mtDNA fragment or of the whole mitochondrial genome can reveal additional phylogeographic structure, in particular among recently diverged lineages [58-63]. Such a larger fragment could help overcome some of the topological uncertainties present in our dataset.

Microsatellite markers have been used to study the fine-scale population structure in geographically restricted red fox populations [24,52-54]. Frati et al. [48] used allozymes and cytochrome b sequences to compare genetic variability among some European red fox populations. To date, most studies on the large-scale population structuring and phylogeography of red foxes have utilized mtDNA sequences [21-27,29]. As a maternally inherited molecule with a high mutation rate, compared to the nuclear genome, and fast coalescence (due to lower effective population size than autosomal loci), mtDNA has been used to resolve phylogeographic structures in many taxa. However, as male-mediated gene flow cannot be detected from mtDNA, more complete inferences of the phylogeographic history of a species should include biparentally or paternally inherited markers (e.g., [64,65]), especially in species like the red fox where males disperse more than females [20].

Therefore, the application of SNP chips, modern high-throughput sequencing techniques [63], and/or the establishment of new (nuclear) markers in a synthesis with phenotypic features and ecological adaptations (e.g., [12,66]) will lead to a deeper understanding of the phylogeographic history and adaptations of this widespread generalist.

## Methods

### Samples and DNA extraction

In total, 52 red fox samples and one arctic fox sample were used in this study (Figure 1, Additional file 1). 33 red fox muscle and skin samples were collected in Germany, Finland, Poland, and central Siberia. The arctic fox muscle sample was collected in central Siberia (Additional file 1). All samples originated from dead wildlife legally hunted during hunting season according to local law, and the animals were not killed specifically for this study. No ethical approval or permit for animal experimentation was required. Additionally, 16 hair and 3 fecal samples were collected in Germany.

Total DNA was extracted from muscle and skin samples using a standard salt extraction protocol (modified from the Puregene™ DNA extraction kit). We extracted DNA from hair using the QIAamp DNA Investigator kit (Qiagen, Hilden) protocols for hair and the QIAamp DNA Stool Mini Kit for fecal samples following the manufacturers’ instructions.

### Amplification and sequencing

A 449 bp fragment (excluding primers) from the 5′ end of the mitochondrial control region was amplified using the primers *Vv.CRS1F* 5′-CCCCAAGACTCAAGGAAGAGGCAC and *Vv.CRS1R* 5′-ACACCACAGTTATGTGTGATCATGGGC. These primers were newly designed based on an alignment of published *Vulpes* mitochondrial sequence [GenBank:GQ374180, AF098155, EU935091 (unpublished); GenBank:AM181037, NC_008434 [67]; GenBank:AB292765, AB292754, AB292741 [21]; GenBank:D83639 [68]; GenBank:AJ585358 [69]]. The forward primer is located at the end of the mitochondrial tRNA-Thr and the beginning of the tRNA-Pro genes, and the reverse primer is located 397 nucleotides into in the control region. The amplified region encompasses the hypervariable region targeted in previous red fox control region studies, allowing direct comparison of the data. PCR reactions were carried out in 15 μl volumes containing approximately 15 ng of genomic DNA, 0.27 μM of each primer, 0.16 μg/μl BSA (New England Biolabs, Ipswich, MA, USA), and 0.8 × of VWR Taq DNA Polymerase Master Mix containing a final concentration of 1.6 mM MgCl_2_ (VWR, Darmstadt, Germany). PCR was performed on a Unocycler (VWR, Darmstadt, Germany) using the following thermal profile: 3 min at 95°C prior to 40 cycles of 30 s at 94°C, 25 s at 59°C, and 1 min 15 s at 72°C; followed by an extension step of 10 min at 72°C. PCR products were detected using standard 1.5% agarose gel electrophoresis, and cycle sequenced with BigDye 3.1 chemistry (Applied Biosystems, Foster City, CA, USA) according to the manufacturer’s recommendation using 1/12^th^ of the reaction mix, with 0.16 μl of BigDye in a 10 μl total volume reaction. Detected PCR products were run on an ABI 3100 instrument (Applied Biosystems). Electropherograms were assembled, checked manually, and sequences were aligned using Geneious 5.4 (Biomatters).

### Sequence analyses

A total of 677 previously published and publicly available sequences from wild red fox populations were collated from GenBank for the control region [21-25,27,28] (Figure 1, Additional file 1). Aubry et al. [23], Sacks et al. [24], and Statham et al. [27] obtained most sequences from museum specimens (1850–1991), the 35 sequences from Teacher et al. [25] were from the Late Pleistocene and early Holocene. All other sequences were of recent origin (>1989) [21,22,24,27,28]. The 52 newly obtained red fox sequences (from samples collected since 2009) were added to a final alignment containing in total 729 red foxes, with a length of 335 bp. Ancient DNA samples from Teacher et al. [25] and one modern sequence from Valière et al. [28] were shorter (179–268 bp), and therefore omitted in network analyses (see below). A comprehensive list of all analyzed sequences, including geographic origins (see also Figure 1), GenBank accession numbers, and referenced study is provided in Additional file 1.

### Data analyses

We determined parameters of within-population variability for all samples and major lineages using Arlequin 3.5 [70], calculating the number of segregating sites and haplotypes, haplotype diversity (Hd), nucleotide diversity (π), and Fu’s F_S_[71], an indicator of population expansion when it is negative and significant (p ≤ 0.02) [35,70,71] (Table 1).

Mismatch distributions under the sudden expansion model [72] were modeled and investigated in Arlequin 3.5 [70]. The sudden expansion model assumes population growth from a population at equilibrium with θ = θ_0_ to a new size with θ = θ_1_ within τ units of mutational time, with τ = 2*u*t (u = substitution rate per lineage for the entire DNA fragment, and t = number of generations since the expansion; see [73]). Time since expansion was thus calculated by dividing the estimate of τ by the product of: 335 (sequence length in base pairs) and the divergence rate (twice the per-lineage substitution rate; see [74]) in percent per year. Generation time for red foxes was assumed to be one year [16,75]. To test goodness-of-fit of the observed mismatch distribution to that expected under the sudden population expansion model, the sum of squared deviations [76] was computed in Arlequin with 10,000 replicates. To calculate time since expansion for those lineages that did not deviate from sudden expansion (p > 0.05), we assumed a per-lineage substitution rate of: (i) 7.1% substitutions per lineage per million years (or u = 2.379*10^-5^), a rate previously used for the arctic fox [2], and (ii) 33.2% substitutions per lineage per million years (u = 1.112*10^-4^), the mean rate estimated from two independent simulations in BEAST (*Scenario 1*; details see below) (Table 3).

A median-joining network of nucleotide sequences (n = 693) was constructed using the software Network 4.6.1.0 [77] (Figure 3). Due to their shorter sequence length and thus missing data, all 35 ancient samples from Teacher et al. [25] and one modern sample from Valière et al. [28] were excluded.

To identify the model of sequence evolution that best fit the data, we used jmodeltest 0.0.1 [78], which suggested the TN93 + G + I model of sequence evolution. A phylogeny and divergence time estimates for different lineages were obtained from simulations in BEAST v1.7.5 [79]. For computational reasons, a maximum number of eight individuals was included per haplotype. BEAST was set to run for 1 billion generations, sampling every 10,000^th^ generation. Convergence was checked in Tracer v1.5. Two runs with identical settings were combined before resampling ca. 20,000 trees, both using LogCombiner v1.7.5 (without setting a burn-in). A maximum clade credibility tree was constructed using TreeAnnotator with a burn-in of 10%. Besides the constant population size model, we also evaluated the exponential population growth model implemented in BEAST.

A combined approach was used for dating of phylogeographic events, utilizing the arctic fox as exterior calibration point, plus tip dates based on published ancient DNA red fox sequences [25]. This approach accounts for major discrepancies from the possible time dependency of the molecular clock on recent evolutionary time scales [32]. The fossil tip calibrations are closer to the phylogeographic time frames of interest than the exterior calibration point, avoiding possible rate shifts. To further validate the robustness of our dating methodology, we performed a simulation using only tip dates, without setting a prior for the root height of the tree.

The divergence time to the arctic fox was used as exterior calibration point, because to our knowledge this is the closest relative to the red fox with an available divergence time estimate.

We tested three scenarios in BEAST, all three using the same tip dates, but varying root heights as exterior calibration points (Table 2). *Scenario 1:* the uniform prior for the root height was set to 1.75-4 million years (My), according to the 95% credibility interval in Perini et al. [80] for the divergence time between red and arctic fox (Figure 2). *Scenario 2:* we tested a uniform prior that spanned a period of 5.1-6 My, according to the 95% credibility interval for another divergence time estimate by Nyakatura and Bininda-Emonds [81]. *Scenario 3:* a very recent divergence time scenario was tested, based on the first appearance of the red fox in the fossil record (0.5-1 Mya) [30,31], setting the minimum age of the root height to 0.5 My and using a lognormal prior. As the speciation event very likely happened earlier than 0.5 Mya [80,81], the 95% interval of the lognormal prior included a period of up to 5.9 My.

A phylogenetic analysis of haplotype data was conducted in MrBayes 3.2 [82] (Additional file 3). We used jmodeltest 0.0.1 [78] to find the model of evolution that best fits the data (HKY + G + I). The analysis was run for 15 million MCMC generations sampling every 2,000^th^ generation, with a burnin of 25%. We used four heated chains and confirmed convergence using the potential scale reduction factor [83], a convergence diagnostic implemented in MrBayes, which approached 1.000 for all parameters.

## Competing interests

The authors declare that they have no competing interests.

## Authors’ contributions

VEK performed laboratory analyses, analyzed the data, and wrote the manuscript. AJ helped conceive the study and contributed reagents. NL, NS, AAS, TH, and CN contributed samples. KS contributed samples and extracted non-invasive samples. NL, AJ, NS, AAS, TH, KS, and CN participated in revising the manuscript. FH conceived the study, analyzed the data, and wrote the manuscript. All authors read and approved the final manuscript.

## Supplementary Material

Additional file 1 **Sequence information for all individuals analyzed in this study.** Individual IDs, haplotype frequencies, consecutive haplotype numbers, individuals with identical sequence, geographic origins, age assumed for the BEAST analyses, GenBank accession numbers, corresponding abbreviations used in the original source studies, and the corresponding references are provided. The designation of individuals is explained in detail in the table caption. Click here for file

Additional file 2 **Sample size and number of mtDNA control region haplotypes for newly sequenced red foxes.** This pdf-file contains a table giving details on haplotypes (novelty, haplotype-sharing) that were reconstructed from 52 newly sequenced red foxes. Click here for file

Additional file 3 **Bayesian inference tree of red fox mtDNA control region haplotypes.** This png-file contains a Bayesian inference tree that was based on 175 haplotypes reconstructed in MrBayes. All major lineages are indicated by square brackets. Interesting haplotypes within the Holarctic lineage are indicated as follows: grey stars: Japanese Hokkaido Ia and Ib haplotypes (Holarctic lineage); black stars: Serbian haplotypes; black stars with parallel white stripes: central Siberian haplotypes; black stars with white edge: North American haplotypes (Holarctic lineage). Click here for file
