# Supplementary material for: A range-wide synthesis and timeline for phylogeographic events in the red fox (Vulpes vulpes)
Source: BMC Evol Biol. 2013 Jun 5;13:114. doi: 10.1186/1471-2148-13-114 (PMC3689046; doi:10.1186/1471-2148-13-114)
Supplement: Additional file 1 — Sequence information for all individuals analyzed in this study. Individual IDs, haplotype frequencies, consecutive haplotype numbers, individuals with identical sequence, geographic origins, age assumed for the BEAST analyses, GenBank accession numbers, corresponding abbreviations used in the original source studies, and the corresponding references are provided. The designation of individuals is explained in detail in the table caption. [file 1471-2148-13-114-S1.pdf]

Additional file 2 – Sequence information for all individuals analyzed in this study
Individual IDs, haplotype frequencies, consecutive haplotype numbers, individuals with identical sequence, geographic origins, age assumed for the BEAST analyses, GenBank accession numbers, corresponding abbreviations used in the original source studies, and the corresponding references are provided.

Designation of individuals:

1. Individual IDs include a letter code indicating the geographic origin of the individual:

   RS = Russia, Siberia; **D** = Germany; **F** = Finland; **P** = **POL** = Poland; **Swit** = Switzerland; **Fr** = France; **Ire** = Ireland; **Swe** = Sweden; **Au** = Austria; **Bel** = Belgium; **Lux** = Luxembourg; **Sp** = Spain; **UK** = United Kingdom; **EU** = Europe; **AS** = Asia; **CA** = USA, Southern California; **AK** = USA, Alaska; **WC** = Western Canada; **CC** = Central Canada; **EC** = Eastern Canada; **RM** = USA, Rocky Mountains; **CR** = USA, Cascade Range; **SC** = USA, Southern Cascades; **NC** = USA, Northern Cascades; **ORW** = USA, Western Oregon; **SV** = USA, California, Sacramento Valley; **SJV** = USA, California, San Joaquin Valley; **GB** = USA, Great Basin; **SN** = USA, Sierra Nevada; **WA** = USA, Western Washington; **ES** = Southeastern United States; **CS** = Central United States; **NAm** = North America; **JH** = Japan, Hokkaido; **JHK** = Japan, Honshu / Kyushu; **RP** = Russia, Primorye; **SU** = Serbia, Surcin; **RA** = Serbia, Radojevo; **BD** = Serbia, Banatski Dvor; **BE** = Serbia, Becej; **MO** = Serbia, Mokrin.
2. The second part of the individual ID is a letter or a number code corresponding to the ID of this individual or of this haplotype published on GenBank, or to the lab IDs for the newly sequenced foxes. Ancient samples from Teacher et al. [1] are additionally marked with "_anc".
3. Valière et al. [2], Aubry et al. [3], Sacks et al. [4], Statham et al. [5], Inoue et al. [6], and Kirschning et al. [7] published haplotypes (not individuals) on GenBank and provided haplotype frequencies and geographic origins separately in their papers. Therefore, the second column of our table indicates the frequency of a haplotype found in a certain region.

   In those cases where a haplotype was found more than once an a certain region, we added a third part to the individual ID: We either numbered the corresponding individuals alphabetically (for individuals from Aubry et al. [3], Sacks et al. [4], Statham et al. [5], and Inoue et al. [6]) OR we added a consecutive number (for individuals from Kirschning et al. [7]).

   Examples: Haplotype 4 from Aubry et al. [3] was found three times in Europe: EU4**A**, EU4**B**, and EU4**C**; Haplotype 1 (D1) from Inoue et al. [6] was found 29 times on Hokkaido: JH1**A**, JH1**B**, ..., JH1**Z**, JH1**AA**, JH1**AB**, JH1**AC**. Haplotype A from Kirschning et al. [7] was found four times in Surcin, Serbia: SU_A**1**, SU_A**2**, SU_A**3**, SU_A**4**, and once in Radojevo, Serbia: RA_A**1**.

[a] Identical to other individuals in a 335 bp alignment of 729 sequences.

Individual ID, this study	Frequency	Consecutive haplotype number	Identical individuals[a]	Geographic origin	Age assumed for BEAST analyses	Accession number(s)	ID used in source studies	Reference(s)
RS56	1	1	RS65, RS66, RS68	Russia, Siberia, Longot-egan	0	HF677203	--	this study
RS65	1	1	RS56, RS66, RS68	Russia, Siberia, Panaevsk	0	HF677204	--	this study
RS66	1	1	RS56, RS65, RS68	Russia, Siberia, Panaevsk	0	HF677205	--	this study
RS67	1	2	--	Russia, Siberia, Panaevsk	0	HF677206	--	this study
RS68	1	1	RS56, RS65, RS66	Russia, Siberia, Panaevsk	0	HF677207	--	this study
RS71	1	3	--	Russia, Siberia, Kharbey	0	HF677208	--	this study
D01	1	4	D02, D03, D04, D05, D06, D07	Germany, Hesse, Taunus	0	HF677209	--	this study
D02	1	4	D01, D03, D04, D05, D06, D07	Germany, Hesse, Schwalbach am Taunus	0	HF677210	--	this study
D03	1	4	D01, D02, D04, D05, D06, D07	Germany, Hesse, Schwalbach am Taunus	0	HF677211	--	this study
D04	1	4	D01, D02, D03, D05, D06, D07	Germany, Hesse, Schwalbach am Taunus	0	HF677212	--	this study
D05	1	4	D01, D02, D03, D04, D06, D07	Germany, Hesse, Schwalbach am Taunus	0	HF677213	--	this study
D06	1	4	D01, D02, D03, D04, D05, D07	Germany, Hesse, Flörsheim	0	HF677214	--	this study
D07	1	4	D01, D02, D03, D04, D05, D06	Germany, Hesse, Flörsheim	0	HF677215	--	this study
D08	1	5	POLI67, Swit12	Germany, Hesse, Schwalbach am Taunus	0	HF677216	--	this study
D09	1	6	Fr2, Swit2	Germany, Lüneburger Heide	0	HF677217	--	this study
D10	1	7	D417, D418, D650, D652, D654, D657, D664, D665	Germany, Lüneburger Heide	0	HF677218	--	this study
D11	1	8	--	Germany, Lüneburger Heide	0	HF677219	--	this study
D110	1	9	D506	Germany, Thuringia, Breiter Berg	0	HF677220	--	this study

Individual ID, this study	Frequency	Consecutive haplotype number	Identical individuals[a]	Geographic origin	Age assumed for BEAST analyses	Accession number(s)	ID used in source studies	Reference(s)
D284	1	10	D582	Germany, Harz, Kyffhäuser	0	HF677221	--	this study
D506	1	9	D110	Germany, Thuringia, Bettenhausen	0	HF677222	--	this study
D582	1	10	D284	Germany, Rhineland-Palatinate, Gödenroth	0	HF677223	--	this study
D408	1	11	D655, D660, POLI68	Germany, Lower Saxony, Elm	0	HF677224	--	this study
D417	1	7	D10, D418, D650, D652, D654, D657, D664, D665	Germany, Hesse, National Park Kellerwald	0	HF677225	--	this study
D418	1	7	D10, D417, D650, D652, D654, D657, D664, D665	Germany, Hesse, National Park Kellerwald	0	HF677226	--	this study
D650	1	7	D10, D417, D418, D652, D654, D657, D664, D665	Germany, Hesse, National Park Kellerwald	0	HF677227	--	this study
D652	1	7	D10, D417, D418, D650, D654, D657, D664, D665	Germany, Hesse, National Park Kellerwald	0	HF677228	--	this study
D654	1	7	D10, D417, D418, D650, D652, D657, D664, D665	Germany, Hesse, National Park Kellerwald	0	HF677229	--	this study
D655	1	11	D408, D660, POLI68	Germany, Hesse, National Park Kellerwald	0	HF677230	--	this study
D657	1	7	D10, D417, D418, D650, D652, D654, D664, D665	Germany, Hesse, National Park Kellerwald	0	HF677231	--	this study
D658	1	12	EU57, Swit11	Germany, Hesse, National Park Kellerwald	0	HF677232	--	this study

Individual ID, this study	Frequency	Consecutive haplotype number	Identical individuals[a]	Geographic origin	Age assumed for BEAST analyses	Accession number(s)	ID used in source studies	Reference(s)
D660	1	11	D408, D655, POLI68	Germany, Hesse, National Park Kellerwald	0	HF677233	--	this study
D664	1	7	D10, D417, D418, D650, D652, D654, D657, D665	Germany, Hesse, National Park Kellerwald	0	HF677234	--	this study
D665	1	7	D10, D417, D418, D650, D652, D654, D657, D664	Germany, Hesse, National Park Kellerwald	0	HF677235	--	this study
D067	1	13	D068, D069	Germany, Hesse	0	HF677236	--	this study
D068	1	13	D067, D069	Germany, Hesse	0	HF677237	--	this study
D069	1	13	D067, D068	Germany, Hesse	0	HF677238	--	this study
F01	1	14	--	Finland, Kronoby, Ostrobothnia	0	HF677239	--	this study
P01	1	15	--	Poland, Carpathians, Tatra National Park, 5 Stawów valley	0	HF677240	--	this study
P02	1	16	POLI69	Poland, Carpathians, Tatra National Park, Chocholowska valley	0	HF677241	--	this study
POLI67	1	5	D08, Swit12	Poland, Voivodeship Podlaskie, Białowieża Forest	0	HF677242	--	this study
POLI68	1	11	D408, D655, D660	Poland, Voivodeship Podlaskie, Drohiczyn	0	HF677243	--	this study
POLI69	1	16	P02	Poland, Voivodeship Warmińsko - Mazurskie, Samplawa	0	HF677244	--	this study
POLI71	1	17	--	Poland, Voivodeship Podlaskie, Klukowo, Szepietowo	0	HF677245	--	this study
POLI72	1	18	--	Poland, Voivodeship Podlaskie, Klukowo, Szepietowo	0	HF677246	--	this study

Individual ID, this study	Frequency	Consecutive haplotype number	Identical individuals[a]	Geographic origin	Age assumed for BEAST analyses	Accession number(s)	ID used in source studies	Reference(s)
POLI74	1	19	--	Poland, Voivodeship Podlaskie, Klukowo, Szepietowo	0	HF677247	--	this study
POLI75	1	20	--	Poland, Voivodeship Podlaskie, Klukowo, Szepietowo	0	HF677248	--	this study
POLI76	1	21	--	Poland, Voivodeship Podlaskie, Klukowo, Szepietowo	0	HF677249	--	this study
POLI77	1	22	--	Poland, Carpathians, Bieszczady Mountains	0	HF677250	--	this study
POLI78	1	23	--	Poland, Carpathians, Bieszczady Mountains	0	HF677251	--	this study
POLI79	1	24	POLI81	Poland, Carpathians, Bieszczady Mountains	0	HF677252	--	this study
POLI80	1	25	--	Poland, Carpathians, Bieszczady Mountains	0	HF677253	--	this study
POLI81	1	24	POLI79	Poland, Carpathians, Bieszczady Mountains	0	HF677254	--	this study
Arctic fox	1	--	--	Russia, Siberia, Longot-egan	0	HF677255	--	this study
Fr1	1	26	Fr3	France, Drome	0	af338789	F1	[1, 2]
Fr2	1	6	D09, Swit2	France, Isere, Savoie, Hautes Alpes	0	af338790	F2	[1, 2]
Swit2	1	6	D09, Fr2	Switzerland, Valais and Fribourg	0	af338790	F2	[1, 2]
Fr3	1	26	Fr1	France, Hautes Alpes and Savoie	0	af338791	F3	[1, 2]
Fr4	1	27	Swit4	France, Savoie	0	af338792	F4	[2, 1]
Swit4	1	27	Fr4	Switzerland, Valais	0	af338792	F4	[1, 2]
Fr5	1	28	--	France, Alpes de Haute Provence	0	af338793	F5	[1, 2]
Fr6	1	29	--	France, Pyrenees Orientales	0	af338794	F6	[1, 2]

Individual ID, this study	Frequency	Consecutive haplotype number	Identical individuals[a]	Geographic origin	Age assumed for BEAST analyses	Accession number(s)	ID used in source studies	Reference(s)
Fr7	1	30	--	France, Alpes de Haute Provence	0	af338795	F7	[1, 2]
Fr8	1	31	--	France, Isere	0	af338796	F8	[1, 2]
Fr9	1	32	--	France, Alpes de Haute Provence	0	af338797	F9	[1, 2]
Fr10	1	33	--	France, Hautes Alpes	0	af338798	F10	[1, 2]
Swit11	1	12	D658, EU57	Switzerland, Valais	0	af338799	F11	[1, 2]
Swit12	1	5	D08, POLI67	Switzerland, Valais	0	af338800	F12	[1, 2]
Fr13	1	34	--	France, Alpes Maritimes	0	af338801	F13	[1, 2]
Swit14	1	35	--	Switzerland, Graubunden	0	af338802	F14	[1, 2]
Fr15	1	36	--	France, Alpes de Haute Provence	0	af487736	F15	[1, 2]
Fr16	1	37	--	France, Drome	0	af487737	F16	[1, 2]
Fr17	1	38	--	France, Pyrenees Orientales	0	af487738	F17	[1, 2]
Fr18	1	39	--	France, Alpes de Haute Provence	0	af487739	F18	[1, 2]
Fr19	1	40	--	France, Alpes de Haute Provence	0	af487740	F19	[1, 2]
Fr20	1	41	--	France, Hautes Alpes	0	af487741	F20	[1, 2]
Fr21	1	42	--	France, Drome	0	af487742	F21	[1, 2]
Fr21	1	43	--	France, Hautes Alpes	0	af487742	F21	[1, 2]
Fr22	1	44	--	France	0	af487743	F22	[1, 2]
Fr23	1	45	--	France, Isere	0	af487744	F23	[1, 2]
Fr24	1	46	--	France, Isere	0	af487745	F24	[1, 2]
Fr25	1	47	--	France, Savoie	0	af487746	F25	[1, 2]
Swit26	1	48	--	Switzerland, Valais	0	af487752	F26	[1, 2]
Swit27	1	49	--	Switzerland, Valais	0	af487753	F27	[1, 2]
Ire1	1	50	--	Ireland	0	aj585358	--	[8]
Swe1	1	51	--	Sweden	0	am181037	--	[9]

Individual ID, this study	Frequency	Consecutive haplotype number	Identical individuals[a]	Geographic origin	Age assumed for BEAST analyses	Accession number(s)	ID used in source studies	Reference(s)
Au52_anc	1	52	--	Austria, Große Peggauerwandhöhle	30000	JN232481	52	[1]
Au48_anc	1	53	--	Austria, Kleine Peggauerwandhöhle	37500	JN232494	48	[1]
Bel6_anc	1	54	--	Belgium, Goyet	12500	JN232493	6	[1]
Bel11_anc	1	55	--	Belgium, Trou de Chaleux	12500	JN232496	11	[1]
Bel18_anc	1	56	--	Belgium, Trou des Nutons, Furfooz	12500	JN232500	18	[1]
D154_anc	1	57	--	Germany, Brillenhöhle, Blaubeuren	14000	JN232490	154	[1]
D153_anc	1	58	--	Germany, Brillenhöhle, Blaubeuren	25000	JN232502	153	[1]
D20_anc	1	59	--	Germany, Steeten/Lahn	25000	JN232505	20	[1]
Fr66_anc	1	60	--	France, Aven des Planes, Monieux	5700	JN232484	66	[1]
Fr68_anc	1	61	--	France, Aven du Chat, Coulet	5700	JN232512	68	[1]
Fr73_anc	1	62	--	France, Coulet des Roches	13600	JN232514	73	[1]
Fr71_anc	1	63	--	France, Coulet des Roches	13600	JN232515	71	[1]
Fr67_anc	1	64	UK139_anc	France, Vauloubeau	5700	JN232486	67	[1]
Fr64_anc	1	65	Fr63_anc	France, Mont Ventoux 2	5700	JN232487	64	[1]
Fr113_anc	1	66	--	France, Reilhac	13500	JN232491	113	[1]
Fr70_anc	1	67	--	France, Coulet des Roches	13600	JN232495	70	[1]
Fr69_anc	1	68	--	France, Aven du Chat, Coulet	5700	JN232503	69	[1]
Fr72_anc	1	69	--	France, Coulet des Roches	13600	JN232504	72	[1]
Fr62_anc	1	70	--	France, Mont Ventoux 2	5880	JN232506	62	[1]
Fr63_anc	1	65	Fr64_anc	France, Mont Ventoux 2	5700	JN232510	63	[1]
Lux105_anc	1	71	--	Luxembourg, Oetrange	50000	JN232488	105	[1]
Lux104_anc	1	72	--	Luxembourg, Oetrange	50000	JN232498	104	[1]
POL150_anc	1	73	--	Poland, Komarowa cave, Poland	12800	JN232489	150	[1]

**Individual ID, this study**	**Frequency**	**Consecutive haplotype number**	**Identical individuals[a]**	**Geographic origin**	**Age assumed for BEAST analyses**	**Accession number(s)**	**ID used in source studies**	**Reference(s)**
POL151_anc	1	74	--	Poland, Mamutowa cave, Poland	21500	JN232497	151	[1]
Sp160_anc	1	75	--	Spain, Can Roqueta II	1500	JN232507	160	[1]
Swit101_anc	1	76	--	Switzerland, Twann	3300	JN232482	101	[1]
Swit85_anc	1	77	--	Switzerland, Twann	3800	JN232499	85	[1]
Swit89_anc	1	78	--	Switzerland, Twann	3800	JN232509	89	[1]
UK124_anc	1	79	--	England, Chelm's Coombe, Cheddar	11000	JN232483	124	[1]
UK126_anc	1	80	--	England, Gough's Cave, Somerset	12500	JN232485	126	[1]
UK161_anc	1	81	--	UK, Scalby Bay, Scarborough	45000	JN232492	161	[1]
UK120_anc	1	82	--	England, Neale's cavern, Devon	5700	JN232501	120	[1]
UK119_anc	1	83	--	England, Ightham	5700	JN232508	119	[1]
UK139_anc	1	64	Fr67_anc	England, Brixham (near Torquay)	37500	JN232511	139	[1]
UK125_anc	1	84	--	England, Chelm's Coombe, Cheddar	11000	JN232513	125	[1]
EU3	1	85	--	Europe = Sweden, Great Britain, Germany, Italy, or Spain	0	FJ830777	3	[3]
EU4A, EU4B, EU4C	3	86	--	Europe = Sweden, Great Britain, Germany, Italy, or Spain	0	FJ830778	4	[3]
EU5	1	87	--	Europe = Sweden, Great Britain, Germany, Italy, or Spain	0	FJ830779	5	[3]
EU51	1	88	--	Europe = Sweden, Great Britain, Germany, Italy, or Spain	0	FJ830806	51	[3]

Individual ID, this study	Frequency	Consecutive haplotype number	Identical individuals[a]	Geographic origin	Age assumed for BEAST analyses	Accession number(s)	ID used in source studies	Reference(s)
EU56	1	89	--	Europe = Sweden, Great Britain, Germany, Italy, or Spain	0	FJ830811	56	[3]
EU57	1	12	D658, Swit11	Europe = Sweden, Great Britain, Germany, Italy, or Spain	0	FJ840491	57	[3]
AS48	1	90	--	Asia = Mongolia	0	FJ830803	48	[3]
AS49	1	91	--	Asia = eastern Siberia	0	FJ830804	49	[3]
AS54	1	92	--	Asia = eastern Siberia	0	FJ830809	54	[3]
AS55A, AS55B, AS55C	3	93	--	Asia = eastern Siberia	0	FJ830810	55	[3]
AS69	1	94	--	Asia = eastern Siberia	0	FJ830821	69	[3]
AS70A, AS70B, ...AS70F	6	95	JH9	Asia = eastern Siberia	0	FJ830822	70	[3]
CA7	1	96	AK7A-M, WC7A-B, RM7, SJV7A-H	USA, Southern California	0	FJ830780	7	[3–5]
AK7A, AK7B, ...AK7M	13	96	CA7, WC7A-B, RM7, SJV7A-H	USA, Alaska	0	FJ830780	7	[3–5]
WC7A, WC7B	2	96	CA7, AK7A-M, RM7, SJV7A-H	Western Canada	0	FJ830780	7	[3–5]
RM7	1	96	CA7, AK7A-M, WC7A-B, SJV7A-H	USA, Rocky Mountains	0	FJ830780	7	[3–5]
SJV7A, SJV7B, ...SJV7H	8	96	CA7, AK7A-M, WC7A-B, RM7	USA, California, San Joaquin Valley	0	FJ830781	7	[3–5]
CA38A, CA38B, ...CA38C	3	97	ES38A-B, GB38, RM38, SJV38A-D, WA38A-L, WC38A-B	USA, Southern California	0	FJ830794	38	[3–5]

Individual ID, this study	Frequency	Consecutive haplotype number	Identical individuals[a]	Geographic origin	Age assumed for BEAST analyses	Accession number(s)	ID used in source studies	Reference(s)
ES38A, ES38B	2	97	CA38A-C, GB38, RM38, SJV38A-D, WA38A-L, WC38A-B	Southeastern United States	0	FJ830794	38	[3–5]
GB38	1	97	CA38A-C, ES38A-B, RM38, SJV38A-D, WA38A-L, WC38A-B	USA, Great Basin	0	FJ830794	38	[3–5]
RM38	1	97	CA38A-C, ES38A-B, GB38, SJV38A-D, WA38A-L, WC38A-B	USA, Rocky Mountains	0	FJ830794	38	[3–5]
SJV38A, AJV38B, ...SJV38D	4	97	CA38A-C, ES38A-B, GB38, RM38, WA38A-L, WC38A-B	USA, California, San Joaquin Valley	0	FJ830794	38	[3–5]
WA38A, WA38B, ...WA38L	12	97	CA38A-C, ES38A-B, GB38, RM38, SJV38A-D, WC38A-B	USA, Western Washington	0	FJ830794	38	[3–5]
WC38A, WC38B	2	97	CA38A-C, ES38A-B, GB38, RM38, SJV38A-D, WA38A-L	Western Canada	0	FJ830794	38	[2–4]
AK39A, AK39B	2	98	--	USA, Alaska	0	FJ830795	39	[3]
WC44	1	99	--	Western Canada	0	FJ830799	44	[3]
WC46	1	100	--	Western Canada	0	FJ830801	46	[3]
AK47A, AK47B, ...AK47D	4	101	--	USA, Alaska	0	FJ830802	47	[3]
AK50	1	102	--	USA, Alaska	0	FJ830805	50	[3]
AK53A, AK53B, AK53C	3	103	--	USA, Alaska	0	FJ830808	53	[3]

Individual ID, this study	Frequency	Consecutive haplotype number	Identical individuals[a]	Geographic origin	Age assumed for BEAST analyses	Accession number(s)	ID used in source studies	Reference(s)
AK58	1	104	--	USA, Alaska	0	FJ830812	58	[3]
CS61	1	105	AK61A-F	Central United States	0	FJ830815	61	[3, 5]
AK61A, AK61B, ...AK61F	6	105	CS61	USA, Alaska	0	FJ830815	61	[3, 5]
AK62A, AK62B	2	106	--	USA, Alaska	0	FJ830816	62	[3]
AK64	1	107	--	USA, Alaska	0	FJ830818	64	[3]
AK71	1	108	--	USA, Alaska	0	FJ830823	71	[3]
WC73A, WC73B, ...WC73N	14	109	EC73, SC73	Western Canada	0	FJ830824	73	[3–5]
EC73	1	109	WC73A-N, SC73	Eastern Canada	0	FJ830824	73	[3–5]
SC73	1	109	EC73, WC73A-N	USA, Southern Cascades	0	FJ830824	73	[3–5]
AK74	1	110	--	USA, Alaska	0	FJ830825	74	[3]
AK75A, AK75B, ...AK75F	6	111	--	USA, Alaska	0	FJ830826	75	[3]
AK77A, AK77B	2	112	--	USA, Alaska	0	FJ830827	77	[3]
AK78A, AK78B, ...AK78D	4	113	--	USA, Alaska	0	FJ830828	78	[3]
ES9A, ES9B, ES9C	3	114	CS9A-B, GB9A-B, WA9A-F, ORW9, CA9A-B, AK9A-B, WC9, CC9, EC9A-G, SJV9A-K	Southeastern United States	0	FJ830781	9	[3–5]

Individual ID, this study	Frequency	Consecutive haplotype number	Identical individuals[a]	Geographic origin	Age assumed for BEAST analyses	Accession number(s)	ID used in source studies	Reference(s)
CS9A, CS9B	2	114	ES9A-C, GB9A-B, WA9A-F, ORW9, CA9A-B, AK9A-B, WC9, CC9, EC9A-G, SJV9A-K	Central United States	0	FJ830781	9	[3–5]
GB9A, GB9B	2	114	ES9A-C, CS9A-B, WA9A-F, ORW9, CA9A-B, AK9A-B, WC9, CC9, EC9A-G, SJV9A-K	USA, Great Basin	0	FJ830781	9	[3–5]
WA9A, WA9B, ...WA9F	6	114	ES9A-C, CS9A-B, GB9A-B, ORW9, CA9A-B, AK9A-B, WC9, CC9, EC9A-G, SJV9A-K	USA, Western Washington	0	FJ830781	9	[3–5]
ORW9	1	114	ES9A-C, CS9A-B, GB9A-B, WA9A-F, CA9A-B, AK9A-B, WC9, CC9, EC9A-G, SJV9A-K	USA, Western Oregon	0	FJ830781	9	[3–5]
CA9A, CA9B	2	114	ES9A-C, CS9A-B, GB9A-B, WA9A-F, ORW9, AK9A-B, WC9, CC9, EC9A-G, SJV9A-K	USA, Southern California	0	FJ830781	9	[3–5]

Individual ID, this study	Frequency	Consecutive haplotype number	Identical individuals[a]	Geographic origin	Age assumed for BEAST analyses	Accession number(s)	ID used in source studies	Reference(s)
AK9A, AK9B	2	114	ES9A-C, CS9A-B, GB9A-B, WA9A-F, ORW9, CA9A-B, WC9, CC9, EC9A-G, SJV9A-K	USA, Alaska	0	FJ830781	9	[3–5]
WC9	1	114	ES9A-C, CS9A-B, GB9A-B, WA9A-F, ORW9, CA9A-B, AK9A-B, CC9, EC9A-G, SJV9A-K	Western Canada	0	FJ830781	9	[3–5]
CC9	1	114	ES9A-C, CS9A-B, GB9A-B, WA9A-F, ORW9, CA9A-B, AK9A-B, WC9, EC9A-G, SJV9A-K	Central Canada	0	FJ830781	9	[3–5]
EC9A, EC9B, ...EC9G	7	114	ES9A-C, CS9A-B, GB9A-B, WA9A-F, ORW9, CA9A-B, AK9A-B, WC9, CC9, SJV9A-K	Eastern Canada	0	FJ830781	9	[3–5]
SJV9A, SJV9B, ...SJV9K	11	114	ES9A-C, CS9A-B, GB9A-B, WA9A-F, ORW9, CA9A-B, AK9A-B, WC9, CC9, EC9A-G	USA, California, San Joaquin Valley	0	FJ830781	9	[3–5]
CS12A, CS12B	2	115	CA12A-E, WC12, CC12A-B, SJV12A-B	Central United States	0	FJ830782	12	[3–5]

Individual ID, this study	Frequency	Consecutive haplotype number	Identical individuals[a]	Geographic origin	Age assumed for BEAST analyses	Accession number(s)	ID used in source studies	Reference(s)
CA12A, CA12B, ...CA12E	5	115	CS12A-B, WC12, CC12A-B, SJV12A-B	USA, Southern California	0	FJ830782	12	[3–5]
WC12	1	115	CS12A-B, CA12A-E, CC12A-B, SJV12A-B	Western Canada	0	FJ830782	12	[3–5]
CC12A, CC12B	2	115	CS12A-B, CA12A-E, WC12, SJV12A-B	Central Canada	0	FJ830782	12	[3–5]
SJV12A, SJV12B	2	115	CS12A-B, CA12A-E, WC12, CC12A-B	USA, California, San Joaquin Valley	0	FJ830782	12	[3–5]
GB17A, GB17B	2	116	EC17A-E	USA, Great Basin	0	FJ830783	17	[3, 5]
EC17A, EC17B, ...EC17E	5	116	GB17A-B	Eastern Canada	0	FJ830783	17	[3, 5]
EC45	1	117	--	Eastern Canada	0	FJ830800	45	[3]
WC60	1	118	--	Western Canada	0	FJ830814	60	[3]
ES76A, ES76B, ...ES76H	8	119	--	Southeastern United States	0	HM590005	76	[5]
CC79A, CC79B	2	120	EC79A-B	Central Canada	0	FJ830829	79	[3]
EC79A, EC79B	2	120	CC79A-B	Eastern Canada	0	FJ830829	79	[3]
ES81	1	121	--	Southeastern United States	0	HM590006	81	[5]
SV18	1	122	--	USA, California, Sacramento Valley	0	GQ911200	18	[4]
GB19A, GB19B, ...GB19Q	17	123	WA19, ORW19A-J, WC19, RM19A-AK, CR19A-C, SC19A-AA, SN19A-F, SV19A-AH	USA, Great Basin	0	FJ830784	19	[3–5]

Individual ID, this study	Frequency	Consecutive haplotype number	Identical individuals[a]	Geographic origin	Age assumed for BEAST analyses	Accession number(s)	ID used in source studies	Reference(s)
WA19	1	123	GB19A-Q, ORW19A-J, WC19, RM19A-AK, CR19A-C, SC19A-AA, SN19A-F, SV19A-AH	USA, Western Washington	0	FJ830784	19	[3–5]
ORW19A, ORW19B, ...ORW19J	10	123	GB19A-Q, WA19, WC19, RM19A-AK, CR19A-C, SC19A-AA, SN19A-F, SV19A-AH	USA, Western Oregon	0	FJ830784	19	[3–5]
WC19	1	123	GB19A-Q, WA19, ORW19A-J, RM19A-AK, CR19A-C, SC19A-AA, SN19A-F, SV19A-AH	Western Canada	0	FJ830784	19	[3–5]
RM19A, RM19B, ...RM19AK	37	123	GB19A-Q, WA19, ORW19A-J, WC19, CR19A-C, SC19A-AA, SN19A-F, SV19A-AH	USA, Rocky Mountains	0	FJ830784	19	[3–5]
CR19A, CR19B, CR19C	3	123	GB19(A-Q), WA19, ORW19(A-J), WC19, RM19(A-AK), SC19A-AA, SN19A-F, SV19A-AH	USA, Cascade Range	0	FJ830784	19	[3–5]
SC19A, SC19B, ...SC19AA	27	123	GB19A-Q, WA19, ORW19A-J, WC19, RM19A-AK, CR19A-C, SN19A-J, SV19A-AH	USA, Southern Cascades	0	FJ830784	19	[3–5]

Individual ID, this study	Frequency	Consecutive haplotype number	Identical individuals[a]	Geographic origin	Age assumed for BEAST analyses	Accession number(s)	ID used in source studies	Reference(s)
SN19A, SN19B, ...SN19J	10	123	GB19A-Q, WA19, ORW19A-J, WC19, RM19A-AK, SC19A-AA, CR19A-C, SV19A-AH	USA, Sierra Nevada	0	FJ830784	19	[3–5]
SV19A, SV19B, ...SV19AH	34	123	GB19A-Q, WA19, ORW19A-J, WC19, RM19A-AK, SC19A-AA, CR19A-C, SN19A-J	USA, California, Sacramento Valley	0	FJ830784	19	[3–5]
RM20	1	124	--	USA, Rocky Mountains	0	GQ911201	20	[4]
GB24	1	125	CR24A-H, NC24A-C	USA, Great Basin	0	FJ830785	24	[3–5]
CR24A, CR24B, ...CR24H	8	125	GB24, NC24A-C	USA, Cascade Range	0	FJ830785	24	[3–5]
NC24A, NC24B, NC24C	3	125	GB24, CR24A-H	USA, Northern Cascades	0	FJ830785	24	[3–5]
CR25	1	126	--	USA, Cascade Range	0	FJ830786	25	[3]
WA26A, WA26B, WA26C	3	126	--	USA, Western Washington	0	HM590004	26	[3, 5]
CR28A, CR28B	2	127	--	USA, Cascade Range	0	FJ830787	28	[3]
NC29	1	128	SN29A-D	USA, Northern Cascades	0	FJ830788	29	[3, 4]
SN29A, SN29B, ...SN29D	4	128	NC29	USA, Sierra Nevada	0	FJ830788	29	[3, 4]
SN30	1	129	--	USA, Sierra Nevada	0	FJ830789	30	[3]

Individual ID, this study	Frequency	Consecutive haplotype number	Identical individuals[a]	Geographic origin	Age assumed for BEAST analyses	Accession number(s)	ID used in source studies	Reference(s)
SC32	1	130	SN32A-C	USA, Southern Cascades	0	FJ830790	32	[3, 4]
SN32A, SN32B, SN32C	3	130	SC32	USA, Sierra Nevada	0	FJ830790	32	[3, 4]
RM42A, RM42B	2	131	--	USA, Rocky Mountains	0	FJ830797	42	[3]
GB43A, GB43B	2	132	RM43	USA, Great Basin	0	FJ830798	43	[3, 5]
RM43	1	132	GB43A-B	USA, Rocky Mountains	0	FJ830798	43	[3, 5]
RM52	1	133	--	USA, Rocky Mountains	0	FJ830807	52	[3]
RM59A, RM59B, RM59C	3	134	--	USA, Rocky Mountains	0	FJ830813	59	[3, 4]
SC66	1	135	--	USA, Southern Cascades	0	GQ911203	66	[4]
RM82A, RM82B	2	136	--	USA, Rocky Mountains	0	GU224186	82	[4]
RM83A, RM83B	2	137	--	USA, Rocky Mountains	0	GU224187	83	[4]
ES87	1	138	--	Southeastern United States	0	HM590008	87	[5]
SC34	1	139	SN34A-D	USA, Southern Cascades	0	FJ830791	34	[3, 4]
SN34A, SN34B, ...SN34D	4	139	SC34	USA, Sierra Nevada	0	FJ830791	34	[3, 4]
CS36	1	140	CA36A-F, SN36	Central United States	0	FJ830792	36	[3, 5]
CA36A, CA36B, ...CA36F	6	140	CS36, SN36	USA, Southern California	0	FJ830792	36	[3, 5]
SN36	1	140	CS36, CA36A-F	USA, Sierra Nevada	0	FJ830792	36	[3, 5]
CS37	1	141	RM37A-B	Central United States	0	FJ830793	37	[3, 5]

Individual ID, this study	Frequency	Consecutive haplotype number	Identical individuals[a]	Geographic origin	Age assumed for BEAST analyses	Accession number(s)	ID used in source studies	Reference(s)
RM37A, RM37B	2	141	CS37	USA, Rocky Mountains	0	FJ830793	37	[3, 5]
RM41A, RM41B	2	142	--	USA, Rocky Mountains	0	FJ830796	41	[3, 4]
WC63A, WC63B, WC63C	3	143	CC63A-D, EC63	Western Canada	0	FJ830817	63	[3]
CC63A, CC63B, ...CC63D	4	143	WC63A-C, EC63	Central Canada	0	FJ830817	63	[3]
EC63	1	143	WC63A-C, CC63A-D	Eastern Canada	0	FJ830817	63	[3]
GB65	1	144	RM65	USA, Great Basin	0	GQ911202	65	[4, 5]
RM65	1	144	GB65	USA, Rocky Mountains	0	GQ911202	65	[4, 5]
WC67	1	145	--	Western Canada	0	FJ830819	67	[3]
RM68	1	146	--	USA, Rocky Mountains	0	FJ830820	68	[3]
NAm84	1	147	--	North America	0	HM590007	84	[5]
JH1A, JH1B, ...JH1AC	29	148	--	Japan, Hokkaido	0	AB292741, AB292743, AB292744, AB292746	C1-D1, C2-D1, C3-D1, C5-D1	[6]
JH2	1	149	--	Japan, Hokkaido	0	AB292745	C4-D2	[6]
JH3A, JH3B, JH3C	3	150	--	Japan, Hokkaido	0	AB292742	C1-D3	[6]
JH4	1	151	--	Japan, Hokkaido	0	AB292747	C5-D4	[6]
JH5A, JH5B, ...JH5D	4	152	--	Japan, Hokkaido	0	AB292748	C6-D5	[6]
JH6A, JH6B, ...JH6H	8	153	--	Japan, Hokkaido	0	AB292749	C6-D6	[6]
JH7	1	154	--	Japan, Hokkaido	0	AB292750	C6-D7	[6]

Individual ID, this study	Frequency	Consecutive haplotype number	Identical individuals[a]	Geographic origin	Age assumed for BEAST analyses	Accession number(s)	ID used in source studies	Reference(s)
JH8A, JH8B	2	155	--	Japan, Hokkaido	0	AB292753	C7-D8	[6]
JH9	1	95	AS70	Japan, Hokkaido	0	AB292751	C6-D9	[6]
JH18A, JH18B, ...JH18D	4	156	--	Japan, Hokkaido	0	AB292761, AB292762, AB292765	C12-D18, C13-D18, C14-D18	[6]
JH19	1	157	--	Japan, Hokkaido	0	AB292763	C13-D19	[6]
JH20	1	158	--	Japan, Hokkaido	0	AB292764	C13-D20	[6]
JHK12A, JHK12B, ...JHK12T	20	159	--	Japan, Honshu / Kyushu	0	AB292755	C9-D12	[6]
JHK13A, JHK13B, JHK13C	3	160	--	Japan, Honshu / Kyushu	0	AB292756	C9-D13	[6]
JHK14	1	161	--	Japan, Honshu / Kyushu	0	AB292757	C9-D14	[6]
JHK15	1	162	--	Japan, Honshu / Kyushu	0	AB292758	C10-D15	[6]
JHK16A, JHK16B, JHK16C	3	163	--	Japan, Honshu / Kyushu	0	AB292759	C10-D16	[6]
JHK17	1	164	--	Japan, Honshu / Kyushu	0	AB292760	C11-D17	[6]
RP10A, RP10B	2	165	--	Russia, Primorye	0	AB292752	C6-D10	[6]
RP11	1	166	--	Russia, Primorye	0	AB292754	C8-D11	[6]
SU_A1, SU_A2, ...SU_A4	4	167	RA_A	Serbia, Surcin	0	HF968440	A	[7]
RA_A	1	167	SU_A1-4	Serbia, Radojevo	0	HF968440	A	[7]
BD_B1, BD_B2	2	168	RA_B	Serbia, Banatski Dvor	0	HF968441	B	[7]
RA_B	1	168	BD_B1-2	Serbia, Radojevo	0	HF968441	B	[7]

Individual ID, this study	Frequency	Consecutive haplotype number	Identical individuals[a]	Geographic origin	Age assumed for BEAST analyses	Accession number(s)	ID used in source studies	Reference(s)
SU_C1, SU_C2, ...SU_C14	14	169	BE_C, BD_C	Serbia, Surcin	0	HF968442	C	[7]
BE_C	1	169	SU_C1-14, BD_C	Serbia, Becej	0	HF968442	C	[7]
BD_C	1	169	SU_C1-14, BE_C	Serbia, Banatski Dvor	0	HF968442	C	[7]
SU_D	1	170	BD_D, RA_D	Serbia, Surcin	0	HF968443	D	[7]
BD_D	1	170	SU_D, RA_D	Serbia, Banatski Dvor	0	HF968443	D	[7]
RA_D	1	170	SU_D, BD_D	Serbia, Radojevo	0	HF968443	D	[7]
BE_E	1	171	BD_E, RA_E	Serbia, Becej	0	HF968444	E	[7]
BD_E	1	171	BE_E, RA_E	Serbia, Banatski Dvor	0	HF968444	E	[7]
RA_E	1	171	BE_E, BD_E	Serbia, Radojevo	0	HF968444	E	[7]
SU_F1, SU_F2, ...SU_F12	12	172	BE_F1-5, BD_F1-7, RA_F1-10, MO_F1-13	Serbia, Surcin	0	HF968445	F	[7]
BE_F1, BE_F2, ...BE_F5	5	172	SU_F1-12, BD_F1-7, RA_F1-10, MO_F1-13	Serbia, Becej	0	HF968445	F	[7]
BD_F1, BD_F2, ...BD_F7	7	172	SU_F1-12, BE_F1-5, RA_F1-10, MO_F1-13	Serbia, Banatski Dvor	0	HF968445	F	[7]
RA_F1, RA_F2, ...RA_F10	10	172	SU_F1-12, BE_F1-5, BD_F1-7, MO_F1-13	Serbia, Radojevo	0	HF968445	F	[7]

Individual ID, this study	Frequency	Consecutive haplotype number	Identical individuals[a]	Geographic origin	Age assumed for BEAST analyses	Accession number(s)	ID used in source studies	Reference(s)
MO_F1, MO_F2, ...MO_F13	13	172	SU_F1-12, BE_F1-5, BD_F1-7, RA_F1-10	Serbia, Mokrin	0	HF968445	F	[7]
SU_G	1	173	BE_G1-11, BD_G1-5, RA_G, MO_G1-11	Serbia, Surcin	0	HF968446	G	[7]
BE_G1, BE_G2, ...BE_G11	11	173	SU_G, BD_G1-5, RA_G, MO_G1-11	Serbia, Becej	0	HF968446	G	[7]
BD_G1, BD_G2, ...BD_G5	5	173	SU_G, BE_G1-11, RA_G, MO_G1-11	Serbia, Banatski Dvor	0	HF968446	G	[7]
RA_G	1	173	SU_G, BE_G1-11, BD_G1-5, MO_G1-11	Serbia, Radojevo	0	HF968446	G	[7]
MO_G1, MO_G2, ...MO_G11	11	173	SU_G, BE_G1-11, BD_G1-5, RA_G	Serbia, Mokrin	0	HF968446	G	[7]
BD_H1, BD_H2, BD_H3	3	174	--	Serbia, Banatski Dvor	0	HF968447	H	[7]
SU_I	1	175	--	Serbia, Surcin	0	HF968448	I	[7]

References

1. Teacher AGF, Thomas JA, Barnes I: **Modern and ancient red fox (*Vulpes vulpes*) in Europe show an unusual lack of geographical and temporal structuring, and differing responses within the carnivores to historical climatic change**. *BMC Evol Biol* 2011, **11**:214.

2. Valière N, Fumagalli L, Gielly L, Miquel C, Lequette B, Poulle M-L, Weber J-M, Arlettaz R, Taberlet P: **Long-distance wolf recolonization of France and Switzerland inferred from non-invasive genetic sampling over a period of 10 years**. *Anim Conserv* 2003, **6**:83–92.

3. Aubry KB, Statham MJ, Sacks BN, Perrine JD, Wisely SM: **Phylogeography of the North American red fox: vicariance in Pleistocene forest refugia**. *Mol Ecol* 2009, **18**:2668–2686.

4. Sacks BN, Statham MJ, Perrine JD, Wisely SM, Aubry KB: **North American montane red foxes: expansion, fragmentation, and the origin of the Sacramento Valley red fox**. *Conserv Genet* 2010, **11**:1523–1539.

5. Statham MJ, Sacks BN, Aubry KB, Perrine JD, Wisely SM: **The origin of recently established red fox populations in the United States: translocations or natural range expansions?** *J Mammal* 2012, **93**:52–65.

6. Inoue T, Nonaka N, Mizuno A, Morishima Y, Sato H, Katakura K, Oku Y: **Mitochondrial DNA Phylogeography of the Red Fox (*Vulpes vulpes*) in Northern Japan**. *Zool Sci* 2007, **24**:1178–1186.

7. Kirschning J, Zachos FE, Cirovic D, Radovic IT, Hmwe SS, Hartl GB: **Population Genetic Analysis of Serbian Red Foxes (*Vulpes vulpes*) by Means of Mitochondrial Control Region Sequences**. *Biochem Genet* 2007, **45**:409–420.

8. Statham MJ, Turner PD, O'Reilly C: **Use of PCR amplification and restriction enzyme digestion of mitochondrial D-loop for identification of mustelids in Ireland**. *Ir Nat J* 2005, **28**:1–6.

9. Arnason U, Gullberg A, Janke A, Kullberg M, Lehman N, Petrov EA, Väinölä R: **Pinniped phylogeny and a new hypothesis for their origin and dispersal**. *Mol Phylogenet Evol* 2006, **41**:345–354.
