# Supplementary material for: A range-wide synthesis and timeline for phylogeographic events in the red fox (Vulpes vulpes)
Source: BMC Evol Biol. 2013 Jun 5;13:114. doi: 10.1186/1471-2148-13-114 (PMC3689046; doi:10.1186/1471-2148-13-114)
Supplement: Additional file 2 — Sample size and number of mtDNA control region haplotypes for newly sequenced red foxes. This pdf-file contains a table giving details on haplotypes (novelty, haplotype-sharing) that were reconstructed from 52 newly sequenced red foxes. [file 1471-2148-13-114-S2.pdf]

Additional file 3 – Sample size and number of mtDNA control region haplotypes for newly sequenced red foxes

Geographic region	n	Haplotypes		
		Total	Thereof not previously described	Thereof shared with another geographic region
All newly sequenced samples	52	25	22	n/a
Siberia	6	3	3	-
Poland	15	13	12	2
Germany	30	10	7	4
Finland	1	1	1	-

n indicates sample size (number of individuals)

n/a not applicable
