# Supplementary figures and images for: A range-wide synthesis and timeline for phylogeographic events in the red fox (Vulpes vulpes)
Source: BMC Evol Biol. 2013 Jun 5;13:114. doi: 10.1186/1471-2148-13-114 (PMC3689046; doi:10.1186/1471-2148-13-114)

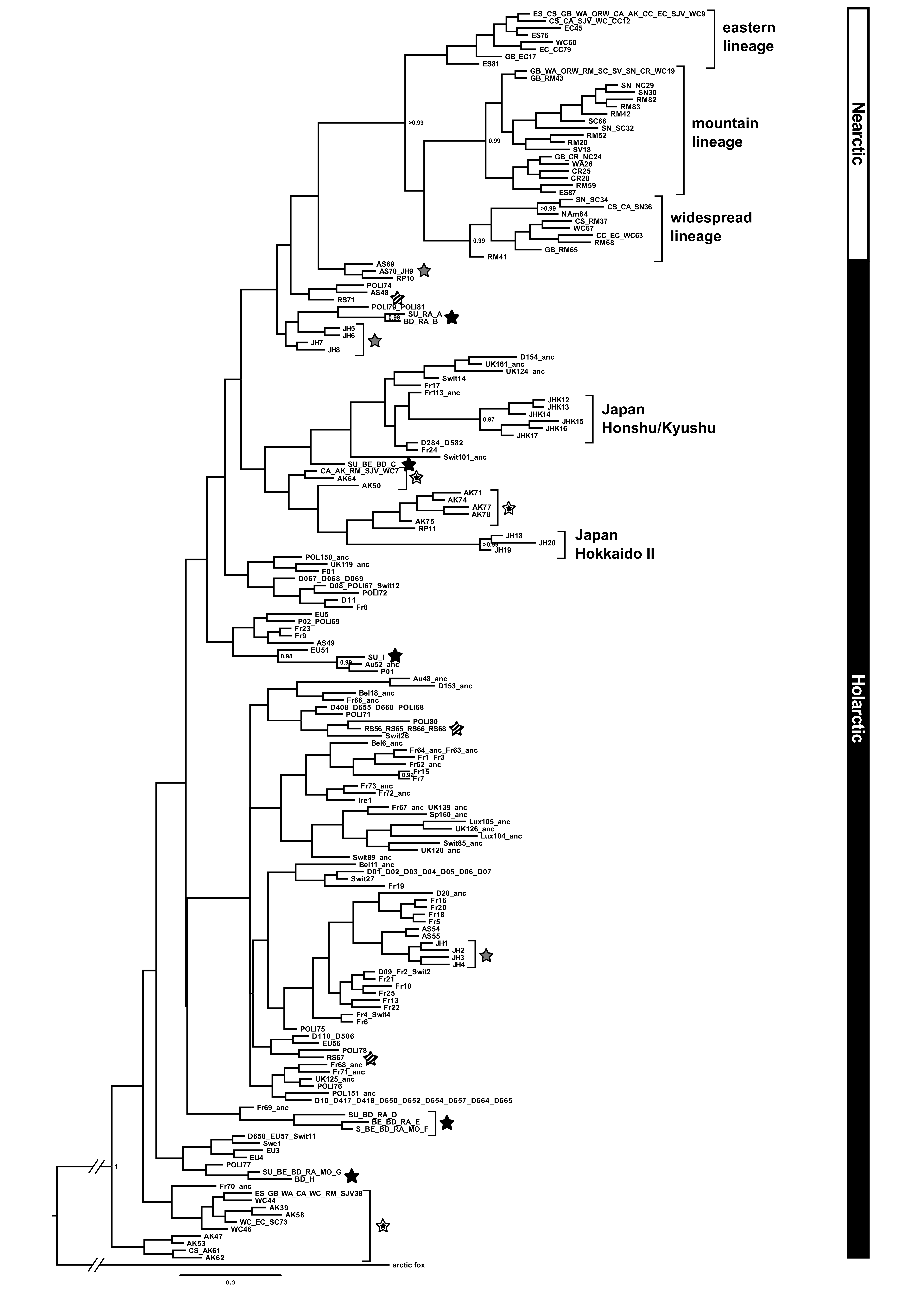

Supplement: Additional file 3 — Bayesian inference tree of red fox mtDNA control region haplotypes. This png-file contains a Bayesian inference tree that was based on 175 haplotypes reconstructed in MrBayes. All major lineages are indicated by square brackets. Interesting haplotypes within the Holarctic lineage are indicated as follows: grey stars: Japanese Hokkaido Ia and Ib haplotypes (Holarctic lineage); black stars: Serbian haplotypes; black stars with parallel white stripes: central Siberian haplotypes; black stars with white edge: North American haplotypes (Holarctic lineage). [file 1471-2148-13-114-S3.png]
